# Integrated Multi-Omics Analysis Reveals the Mechanisms of Intestinal Cell Injury Under Different Levels of Heat Stress

**DOI:** 10.3390/ijms26125798

**Published:** 2025-06-17

**Authors:** Yuchao Feng, Decheng Suo, Ping Gong, Peiling Wei, Lu Zhang, Shu Zhang, Xiaonan Li, Changyuan Wang, Xia Fan

**Affiliations:** 1 State Key Laboratory for Quality and Safety of Agro-Products, Institute of Quality Standards and Testing Technology for Agro-Products, Chinese Academy of Agricultural Sciences, Beijing 100081, China; fengyuchao0321@126.com (Y.F.); suodecheng@caas.cn (D.S.); lixiaonan0507@163.com (X.L.); 2 College of Food, Heilongjiang Bayi Agricultural University, Daqing 163319, China; zshu996@163.com; 3 Institute of Animal Husbandry Quality Standards, Xinjiang Academy of Animal Science, Urumqi 830057, China; gongping2025@126.com (P.G.); weipeiling0830@163.com (P.W.); 4 Center of Agro-Product Safety and Quality of Xinjiang, Urumqi 830006, China; 18290858798@163.com

**Keywords:** heat stress, metabolomics, transcriptomics, mechanisms, pathway

## Abstract

Given the escalating global temperatures and the consequent exacerbation of heat stress, dietary interventions have emerged as a promising therapeutic strategy. The gastrointestinal tract, being exquisitely sensitive to thermal challenges, revealing the underlying mechanisms of intestinal cell injury under high temperature, is essential for developing strategies to prevent heat stress. Here, we integrated metabolomic and transcriptomic analyses to investigate the metabolic and genetic changes in murine intestinal cells in response to different levels of heat stress. The results identified the PI3k-Akt-FoxO pathway as the major heat stress regulatory pathway Kin MODE-K cells. The possible regulatory mechanism is to reduce the expression of the *FoxO* gene through the downstream phosphorylation of PI3K under the stimulation of growth factors such as INS, IGF1 and TGF-β. Then, through acetylation modification, it regulates the expression of the *Gadd45* gene, promotes the expression of *p19* and *BNIP3* genes, and inhibits the expression of the *ATG8* gene, thus inducing apoptosis to remove cells that cannot be repaired. It also promotes *cyclinB*, *PLK*, and Bcl-6 gene expression in cells surrounding apoptotic cells to inhibit apoptosis. It promotes the expression of RAG1/2 to enhance cellular immunity and regulates the *G6pc* gene to maintain the homeostasis of glycogen metabolism and glucose under heat stress. Our findings provide a basis for the regulation of intestinal cell damage due to heat stress through dietary interventions.

## 1. Introduction

With global warming and the frequent occurrence of extreme weather phenomena [1], heat stress is having increasing societal and economic impacts. The effects of heat stress are expected to become even more severe in the future [2]. Excessive exposure to high temperatures during daily activities can have a deleterious health effect. Heat stress is the sum of non-specific response reactions in humans or animals to stimulation by excessively high temperatures that exceed their ability to regulate body temperature [3]. The current threat of global heat stress has drawn attention to the incidence and severity of heat stroke [4]. Heat stroke is a major clinical manifestation of heat stress, characterized by dysfunctional thermoregulation, abnormal protein expression [5], inflammatory damage [6], increased levels of oxidative stress, acid–base imbalance, neuroendocrine alterations, intestinal barrier disruption [7,8], organ disruption and functional impairment [9]. Therefore, the alleviation of the effects of heat stress on human and animal health has been a key research focus in recent years. Specifically, as an important site of nutrient digestion and absorption and the largest immune organ, it is necessary to clarify the mechanisms of heat stress regulation in the small intestine to develop strategies to alleviate heat stress through dietary interventions.

Intestinal tissues are sensitive to heat stress [10], especially during prolonged exposure to high temperature. Heat stress damages the intestinal barrier integrity in animals, resulting in the disruption of tissue and inflammation [11,12]. Heat stress can cause intestinal barrier disruption via several pathways. First, heat stress leads to a compensatory reduction in visceral blood flow, reduces oxygen flow to the intestinal mucosa, promotes oxidative and nitrosative stress, and loosens intestinal epithelial tight junctions. The gut becomes ‘leaky’; the consequent reduction in nutrients and energy to maintain cell viability and function affects cellular metabolism within the intestinal tract, which, in turn, leads to intestinal damage [13]. Second, continuous heat stress induces oxidative stress in intestinal cells. The excess reactive oxygen species generated in this process leads to cellular DNA damage, protein and lipid peroxidation, and, ultimately, apoptosis [14]. Third, heat stress stimulates the secretion of two adipokines, leptin and lipocalin, and the expression of their receptors, leading to a sudden decrease in the amount of voluntary food intake and, hence, additional intestinal mucosal damage [15]. Heat stress can decrease the content of antioxidants and increase the content of pro-oxidants in cells and tissues, which is closely related to intestinal structural and functional damage [16]. While previous studies of the effects of heat stress on intestinal barrier integrity mostly focus on the damage caused by oxidative stress, the underlying mechanisms were not studied in depth.

Numerous studies on the effect of heat stress on cell functions mainly focused on the pathway of cell oxidation, apoptosis, endoplasmic reticulum stress [17,18], as well as the detection of indicators such as immune factors, inflammatory factors [19] and heat shock proteins [20]. In most studies, in vitro physicochemical tests and molecular biology techniques were used [21,22], and heat stress response mechanisms varied among cell sources and types. Furthermore, differences in temperature used to induce heat stress led to inconsistent results. Multi-omics techniques allow for the study of disease mechanisms, identification of disease-causing targets and precision medicine research. The integration of metabolomic–transcriptomic analyses can infer metabolic pathways based on gene expression data, which helps understand how expression changes in metabolic systems affect related biological functions and reflect the effects of external stimuli on biological systems. However, studies on the regulatory mechanisms of heat stress based on multi-omics techniques are scarce. In this study, we investigate the regulation mechanisms of intestinal cells under different levels of heat stress using MODE-K cells using integrated metabolomic and transcriptomic analyses.

## 2. Results

### 2.1. Effects of Different Levels of Heat Stress on Heat Shock Protein 70 Expression, Antioxidant, Tight Junction, Inflammatory Factor, and Survival/Apoptotic Factor mRNA Expression and Mitochondrial Membrane Potential in MODE-K Cells

**Figure 1:**
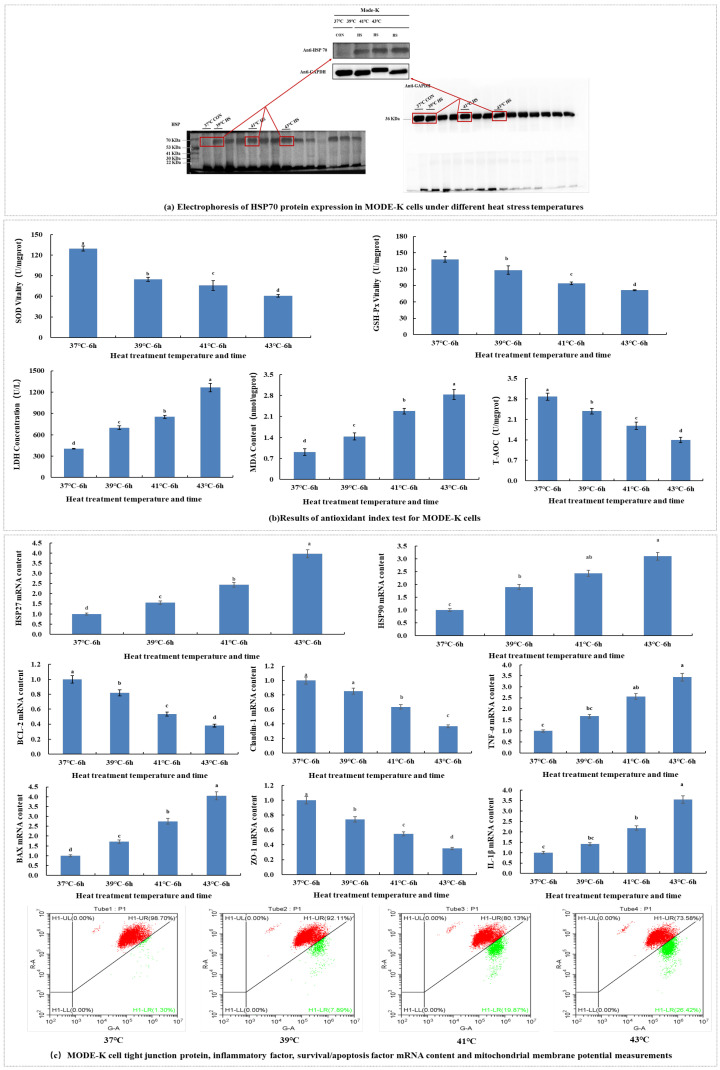
Changes in gene expression of heat shock proteins, tight junctions, antioxidant activity, inflammatory factors, apoptotic factors, and mitochondrial membrane potential in MODE-K cells under different levels of heat stress, as measured by reverse-transcription PCR. In (**a**), CON is the control group and HS is the heat stress group. Letters above the error line in (**b**,**c**) represent differences between pairs of sample groups. Different letters indicate a significant difference (*p* < 0.05), while the presence of the same letter means that the difference is not significant (*p* > 0.05). The percentage in the upper-right corner of the mitochondrial membrane potential represents the percentage of live cells and the percentage in the lower-right corner represents the percentage of apoptotic cells in (**c**), red represents the number of live cells, while green represents the number of apoptotic cells.

Con represents the control group; HS39, HS41 and HS43 represent the 39 °C, 41 °C and 43 °C heat stress treatment groups, respectively. Based on Western blotting, the expression of heat shock protein 70 (HSP70), indicated by the band intensity, increased with increasing levels of heat stress (Figure 1a). The viability of superoxide dismutase (SOD) and glutathione peroxidase (GSH-Px) activities and total antioxidant capacity (T-AOC) significantly decreased (*p* < 0.05) with increasing levels of heat stress. Lactate dehydrogenase (LDH) activity and malondialdehyde (MDA) content significantly increased with increasing levels of heat stress (Figure 1b). The expression of HSP27, HSP70 and HSP90 mRNA significantly increased, while that of ZO-1 and Claudin-1 mRNA levels significantly decreased with increasing level of heat stress. The expression of IL-1β, TNF-α and death regulator Bax mRNA significantly increased, while that of the cell survival factor Bcl-2 significantly decreased with increasing levels of heat stress. The mitochondrial membrane potential gradually increased with increasing levels of heat stress, and the amount of apoptosis gradually increased with approximately 10% (Figure 1c).

### 2.2. Effects of Different Levels of Heat Stress on Metabolites of MODE-K Cells

#### 2.2.1. Metabolite Identification and Principal Component Analysis

**Figure 2:**
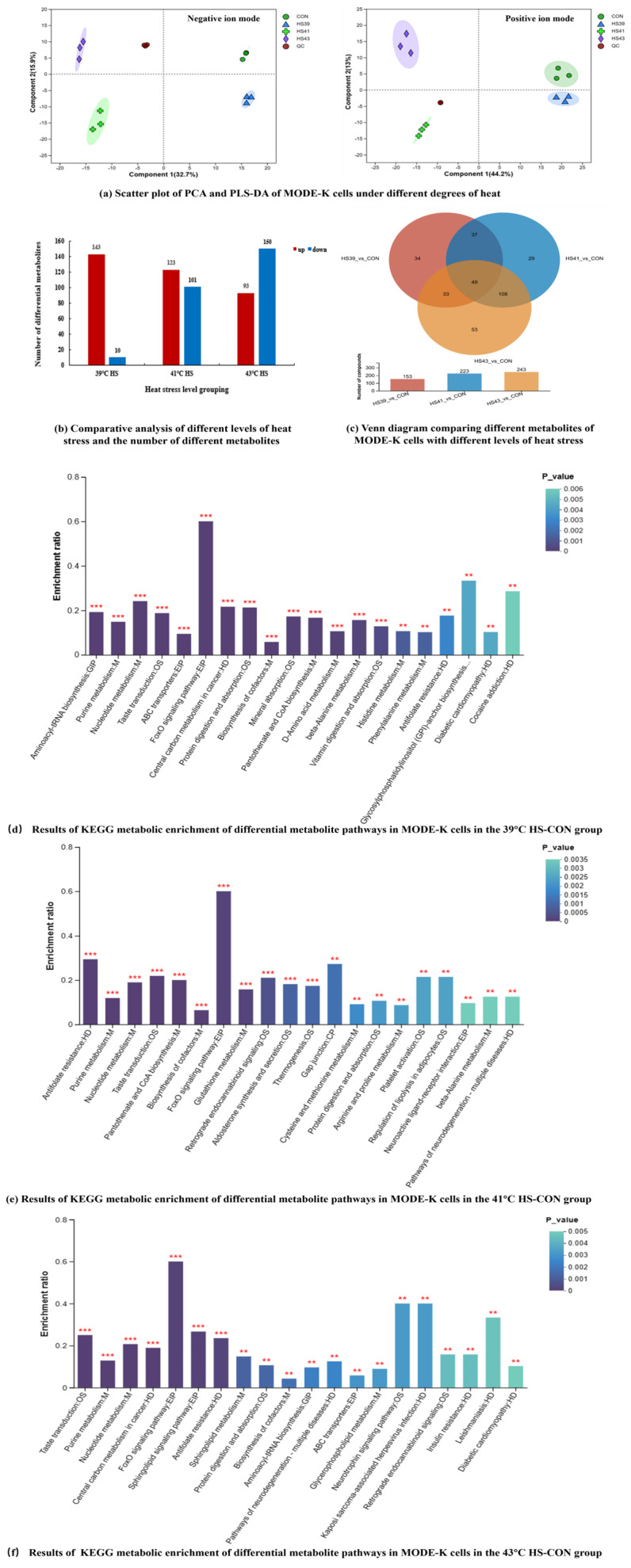
Multivariate analysis results of metabolomics in MODE-K cells under different levels of heat stress. In (**a**), con represents the control group; HS39, HS41 and HS43 represent the 39 °C, 41 °C and 43 °C heat stress treatment groups, respectively; and QC is the quality control sample group. In (**b**), the red bars represent the number of differential metabolites upregulated in content after heat stress, and the blue color represents the amount of downregulation in the content of differential metabolites after heat stress. HS39_vs_CON in (**c**) represents the results of comparative analyses between the 39 °C HS group and the CON group, and the same for the remaining two groups. Numbers in (**c**) represent the number of differential metabolites screened between groups. Asterisks in (**d**–**f**) represent significance level. The more asterisks, the greater the significance, and the color from green to purple means that the significance is decreasing, 0.001 < *p* ≤ 0.01 **, *p* ≤ 0.001 ***.

In total, 735 metabolites were identified in both positive and negative ion modes in the control group (CON) and three HS groups, which were annotated and categorized according to Kyoto Encyclopedia of Genes and Genomes (KEGG) biological functions (Supplementary Figure S1). The cellular metabolites were enriched in lipids (mainly phospholipids and fatty acid substances) and peptides (mainly amino acid substances) in addition to cofactors. Based on orthogonal partial least squares discriminant analysis (OPLS-DA), the profiles of metabolites were clearly distinguished at different temperatures in scatter score plots (Figure 2a).

#### 2.2.2. Screening of Differential Metabolites and KEGG Pathway Enrichment Analysis

Differential metabolites were screened based on a variable importance on projection (VIP) value > 1 and *p* ≤ 0.05 in OPLS-DA. We identified 153, 223 and 243 (Supplementary Tables S1–S3) differential metabolites at 39 °C, 41 °C and 43 °C HS groups, respectively, when compared to the CON group. With increasing temperature, cellular metabolic differences increased, and the number of upregulated differential metabolites gradually decreased, whereas that of downregulated metabolites gradually increased (Figure 2b). Differential metabolites at 39 °C mainly included peptides, amino acids, nucleotides, nucleosides, other nucleic acids, vitamins, cofactors, lipids (glycolipids and phospholipids), and sugars (monosaccharides and oligosaccharides). Differential metabolites at 41 °C and 43 °C were mainly fatty acids, phospholipids, alkanes, other lipids, bases, nucleotides, nucleosides, other nucleic acids, amino acids and peptides. Vitamins were also identified as differential metabolites at 41 °C.

Venn plots of the differential metabolites in the 3 HS groups are shown in Figure 2c. In total, there were 35 differential metabolites in MODE-K cells exposed to different temperatures, which were key metabolic markers of cellular heat stress regulation (Table 1). All of these metabolites were upregulated after heat stress, except for phenylalanine, glycine, valine, hydroxyproline, which were downregulated. These metabolites include amino acids and their derivatives, nucleotides and nucleotide-related substances, other organic acids and their derivatives, peptides, carnitines, phospholipids, substances related to energy metabolism, substances related to carbohydrates, and other types of substances. Table 1 shows heat stress-related metabolic markers in MODE-K cells.

The differential metabolites were analyzed for KEGG pathway enrichment (Figure 2d–f, Supplementary Tables S4–S6). Samples in all three HS groups were significantly enriched for 20 key pathways, with first-order pathways, including metabolism, human disease, organismal systems, genetic information processing, environmental information processing and cellular processes.

The most predominant secondary pathways enriched after heat stress at 39 °C included the FoxO signaling pathway, central carbon metabolism in cancer, protein digestion and absorption, aminoacyl tRNA biosynthesis and ABC transporter proteins. Pathways enriched at 41 °C included pantothenic acid, coenzyme A biosynthesis, taste transduction, purine metabolism, FoxO signaling pathway, glutathione metabolism and thermogenic pathways. Those enriched at 43 °C included central carbon metabolism, taste transduction, purine metabolism, sphingolipid signaling pathway and FoxO signaling pathway. By comparing the key pathways enriched in the three HS groups, five common heat stress pathways were identified (Table 2), including the FoxO signaling pathway, antifolate resistance, taste transduction, protein digestion and absorption, and purine metabolism.

### 2.3. Effects of Different Levels of Heat Stress on the Transcripts of MODE-K Cells

#### 2.3.1. Principal Component Analysis (PCA) and Differential Gene Screening

**Figure 3:**
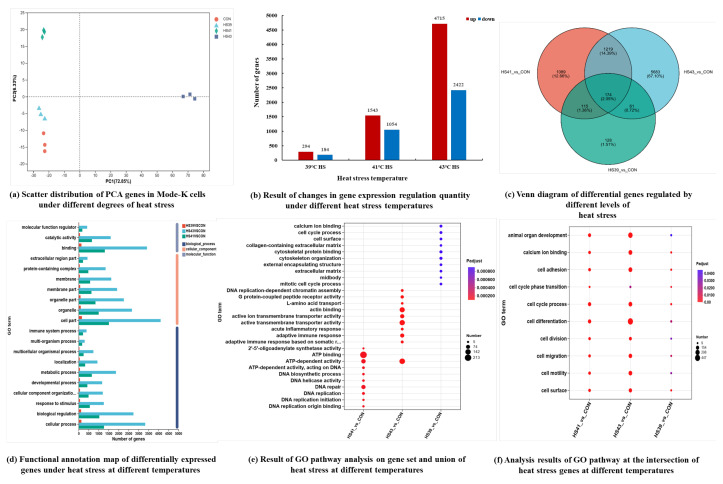
Multivariate analysis results of transcriptomics in MODE-K cells under different levels of heat stress. In (**a**), con represents the control group, and HS39, HS41, and HS43 represent different levels of heat stress at 39 °C, 41 °C, and 43 °C, respectively. In (**b**), red bars represent the number of differential genes whose content was upregulated after heat stress, and blue represents the number of differential genes whose content was downregulated after heat stress. In (**c**), numbers represent the number of differential genes. In (**d**), the vertical axis in the chart represents the secondary GO classification terms, the horizontal axis represents the number of genes/transcripts in the secondary classification compared, and the color represents different gene sets. In (**e**,**f**), the vertical axis represents the pathway/term name, the horizontal axis represents the gene set name, the size of the dots represents the number of genes in this pathway/term, and the color of the dots corresponds to different *p*-value ranges.

We obtained more than 6.04 Gb clean transcriptomic sequencing data for each sample. A sequence comparison of the clean reads of each sample with the reference genome revealed 93.55% to 95.50% similarity. The PCA scatter plots showed that all data points were within the confidence interval, and the different heat stress groups formed distinct clusters (Figure 3a). The distances between the HS groups and the CON group and that among the HS groups increased with increasing temperature.

Differentially expressed genes in the HS groups compared to the CON group were identified based on |log_2_(fold change)| ≥ 1 and *p* < 0.05 (Figure 3b). The expression levels of the upregulated genes were nearly twice as high as those of the downregulated genes. A Venn diagram shows that there were 478, 2597 and 7137 differential genes at 39 °C, 41 °C and 43 °C, respectively (Figure 3c).

#### 2.3.2. Gene Ontology (GO) Functional Annotation and Pathway Enrichment

The results of GO functional annotation (biological process, cellular component, and molecular function) of the differentially expressed genes, GO enrichment analysis, and the intersection of the GO pathway enrichment analysis are shown in Figure 3d–f, respectively. Heat stress mainly affected cell growth and differentiation at 39 °C, affected DNA repair and other processes at 41 °C, and significantly affected transmembrane transport proteins, inflammation and immune reactions at 43 °C.

We found 49 differential key genes under heat stress at 39 °C (Supplementary Table S7). The significantly upregulated genes included *Gm10233*, *Gm11751*, *Gm50092*, *Sall1* and *Igkc*, *Gm49390*. The significantly downregulated genes included *H3c14*, *Pdxp*, *Tmem255a*, *Sorl1*, *Apol10a*, *9230116L04Rik* and *9230116L04Rik*. We found 47 key genes that were altered by heat stress at 41 °C (Supplementary Table S8). The significantly upregulated genes included *Cxcl11*, *Ly6i*, *Gm42845*, *Schlafen4* and *Ccrl2*. The significantly downregulated genes included *Icam2*, *Crybb3*, *Bhlha15*, *Gm13192*, *Apc-ps1*, *9230111E07Rik*, *Gm2244*, *Gm4540*, *Gm42457*, *Rpl10-ps5* and *Gm49599*.

In total, the expression of 254 key genes were altered by heat stress at 43 °C (Supplementary Table S9). The significantly upregulated genes included *Nell2*, *Sema6a*, *Neurog2*, *Kctd19*, *Garin5b*, *Gm17552*, *Tuba3b*, *Lrrc36*, *Gm30593*, *Rmrp*, *0610033M10Rik* and *Muc3a*, et al.

The results of enrichment analysis of all differentially expressed genes to refine the biological processes involved in heat stress regulation are shown in Figure 3e,f, and pathway enrichment information is presented in Supplementary Tables S10–S12. Heat stress at 39 °C mainly affected genes in pathways related to cofactor and vitamin metabolism, digestive system, energy metabolism, endocrine and metabolic diseases, immune system, carbohydrate metabolism, and cell growth and death. Heat stress at 41 °C mainly affected genes related to replication and repair in genetic information and in environmental information processing. Heat stress at 43 °C mainly affected genes in pathways related to cellular processes such as transportation, catabolism, growth and death, and metabolic processes such as amino acid and carbohydrate metabolism.

#### 2.3.3. Metabolomic–Transcriptomic Association Analysis

Annotated Venn diagrams and KEGG pathway analysis results of MODE-K cells under heat stress at 39 °C, 41 °C and 43 °C are shown in Supplementary Figures S2–S4. We found 76, 161 and 172 common pathways annotated in the gene and metabolic sets under heat stress at 39 °C, 41 °C and 43 °C, respectively. There were 249, 449 and 410 matched total differential metabolites, and 199, 1717 and 3291 annotated common differentially expressed genes, respectively. The numbers of differential genes and metabolites in the AMPK, mTOR, FoxO, PI3K-Akt and cAMP signaling pathways, neuroactive ligand–receptor interactions, ABC transporters, and cGMP-PKG and phospholipase D signaling pathways in the classification of environmental information processing were all higher than those in other pathways.

**Figure 4:**
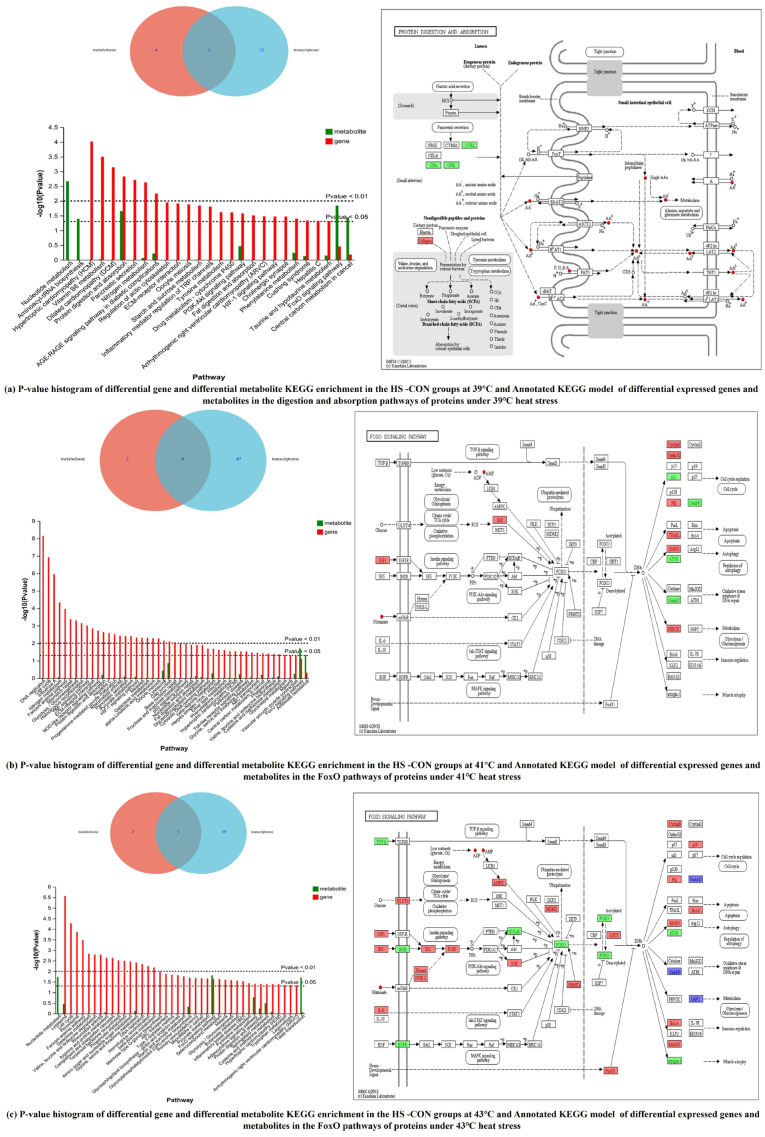
Differentially expressed genes and metabolites at different levels of heat stress and KEGG pathway enrichment. The numbers in the Venn diagram circles represent the number of differential pathways. The circle in the figure indicates metabolites, the box indicates genes, red represents upregulation of metabolites or genes, green represents downregulation of metabolites or genes, and blue represents upregulation or downregulation of metabolites or genes in the KEGG pathway model.

The KEGG pathway enrichment analysis results are shown in Figure 4. The left side of the figure shows the enriched pathways with significant changes in Mode-k cell metabolome or transcriptome under different heat stress temperatures. On the right side is a KEGG model map of the most critical pathways with significant changes in both the metabolome and transcriptome. The figure clearly shows the genes and metabolites regulated in this pathway and their regulation under different temperatures. The total numbers of pathways enriched in significantly altered (*p* < 0.05) gene and metabolic sets under heat stress at 39 °C, 41 °C and 43 °C were 27, 49 and 42, respectively. Detailed information is provided in Supplementary Tables S13–S15. The pathways enriched in significantly (*p* < 0.05) and extremely significantly (*p* < 0.01) differential metabolic and transcriptional sets are the key pathways that affect MODE-K cells under different heat stress temperatures.

The most critical pathway differentially regulated in MODE-K cells in 39 °C HS groups was protein digestion and absorption. KEGG pathway enrichment of differential genes and metabolites in the 39 °C HS group versus the CON group is shown in Figure 4a. Further, 7 differential genes and 12 differential metabolites in the pathway were annotated. *Cpb1*, *Ctrl*, *Cpa2* and *Col11a2* were significantly downregulated in the 39 °C HS group, whereas *Col8a1*, *Col3a1* and *Col1a1* were significantly upregulated. Also, 12 key metabolites, all of which were amino acids, were upregulated in the 39 °C HS group (Table 3). The results of correlation analysis of the 7 differential genes and 12 differential metabolites are shown in Supplementary Table S16. Three differential genes were significantly correlated with nine differential metabolites. *Col8a1* and *Col1a1* were significantly upregulated, and *Col11a2* was significantly downregulated in the protein digestion and uptake pathway in the 39 °C HS group. The metabolites L-valine, L-lysine, L-proline, L-glutamate, L-tryptophan, L-histidine, L-phenylalanine, L-tyrosine and L-glutamate in this pathway were significantly upregulated.

The FoxO signalling pathway, lysosomes, gap junctions, and stress resistance pathway were relatively highly enriched in differential metabolic and gene sets in the 41 °C HS group. The FoxO signalling pathway was the most enriched, as shown in Figure 4b. There were 17 differentially expressed genes and 5 differentially expressed metabolites in this pathway. *Prkag3*, *Igf1*, *Il6*, *Plk4, Ccng2*, *Plk1*, *Ccnb2*, *Tnfsf10*, *Pck2*, *Ccnb1*, *Mapk10*, *Bnip3* and *Prmt1* were significantly upregulated, whereas *Cdkn1*, *Gabarapl1*, *Gabarapl2* and *Gadd45a* were significantly downregulated in the 41 °C HS group, with Tnfsf10 being upregulated nearly 500-times. The five key metabolites were all upregulated (Table 4).

The results of correlation analysis of the 17 differential genes and 5 differential metabolites in the 41 °C HS group are shown in Supplementary Table S17. Additionally, six genes and five metabolites were significantly positively correlated. Key genes in the FoxO signaling pathway, *Pck2*, *Bnip3* and *Prmt* were significantly upregulated, whereas *Gabarapl1*, *Gadd45a* and *Cdkn1a* were significantly downregulated. This was accompanied by a significant upregulation in the levels of L-glutamate, adenosine monophosphate (AMP), adenosine diphosphate (ADP), adenosine-5′-monophosphate (5′-AMP) and L-glutamate.

The FoxO signaling pathway was the most critical pathway modulated in the 43 °C HS group (Table 5). Further, 40 differential genes and 5 differential metabolites were annotated in this pathway. *Gadd45g*, *Cdkn2d*, *Bcl6*, *Irs2*, *Plk1*, *Rag1*, *Agap2*, *G6pc*, *Sirt1* and *Sgk3* et al. were significantly upregulated, and *Gadd45a*, *Insr*, *Ikbkb*, *Foxo4*, *Gabarapl1*, *Tgfb3*, *Fbxo25*, *G6pc3*, *Egfr* and *FBXO25_32* were significantly downregulated (Figure 4c).

## 3. Discussion

Heat stress induces the accumulation of HSPs, which are markers of heat tolerance in animal and cellular models and tissue injury [23,24]. Our results confirmed that heat stress was induced in MODE-K cells in all three HS groups, reflected by the significant changes in the SOD, GSH-PX, and LDH activities and the T-AOC and MDA content, indicating decreased antioxidant contents. The elevated levels of pro-oxidants indicated oxidative damage to the cells and cell membrane integrity disruption, in line with previous findings [16,25,26]. The significant decrease in the expression of the tight junction proteins ZO-1 and claudin-1 indicated that cell polarity, permeability, and barrier function were affected. Heat stress could weaken the immune status of the body [27] and cause oxidative stress, which, in turn, activates various transcription factors, leading to the differential expression of inflammatory genes [28,29,30]. This was confirmed by the significantly increased mRNA levels of IL-1β and TNF-α under heat stress in our study. Heat stress can also lead to apoptosis. With increasing temperature, we observed a significant decrease in the BCL-2 content and significant increases in the Bax content and mitochondrial membrane potential. This suggested that heat stress caused MODE-K cells to undergo apoptosis, which is in line with previous findings [19,31,32]. The variation degree in the above markers at 39 °C, 41 °C and 43 °C confirmed that different temperatures have different effects on intestinal cells.

Studies on heat stress response mechanisms reported to date focused on signaling pathways, such as AMPK/Sirt1-PGC-1α, Keap1-Nrf2-ARE, NF-κB, PI3K/Akt, MAPK and MAPK-Nrf2 [19,33,34,35], which are mostly oxidative stress- and inflammation-related pathways. The FoxO protein signaling pathway regulates cellular responses to environmental signals. Research reports on FoxO signaling in heat stress are scarce. Yoshihara et al. [36] showed that heat stress induced *FoxO3a* phosphorylation in rat skeletal muscle, which was associated with the upregulation of *Hsp72* and activation of the PTEN/Akt and MEK/ERK pathways. During apoptosis, *FoxO* is involved in mitochondria-dependent and mitochondria-independent processes that trigger the expression of death receptor ligands (e.g., Fas ligand, TNF apoptosis ligand, and Bcl-XL) [37]. Zuo et al. [38] found that curcumin upregulated the expression of histone deacetylase 1 via the FoxO signaling pathway, revealing the mechanisms by which induced oxidative stress induces histone deacetylation in human gastric cancer cells. The results of this study showed that heat stress caused significant changes in both the gene set and metabolic set, and KEGG enrichment analysis revealed that the signaling pathways regulated by the FoxO pathway included TGF-β, PI3K-Akt, JAK-STAT, and MAPK, among which the PI3k-Akt-FoxO pathway was the major heat stress regulatory pathway in MODE-K cells (Figure 4). The possible regulatory mechanism is that the downstream phosphorylation of phosphatidylinositol 3-kinase (PI3K) under the stimulation of various growth factors, such as insulin (INS), insulin-like growth factor-1 (IGF1), and transforming growth factor-β (TGF-β), results in the translocation of FoxO proteins from the nucleus to cytoplasm and a reduction in the expression of *FoxO* target genes. The downstream target genes are then regulated by acetylation modification, which is mainly manifested in regulating the expression of the environmental stress protein *Gadd45* gene, activating oxidative stress, promoting the expression of cell cycle inhibitor *p19*, inhibiting cell proliferation, promoting the expression of mitochondrial pro-apoptotic protein *BNIP3*, promoting apoptosis, inhibiting the expression of autophagy gene *ATG8*, and attenuating cellular autophagy, thus inducing apoptosis to clear the cells that can not be repaired. At the same time, these apoptotic cells release signals to promote the expression of cyclin B and serine/threonine protein kinase (PLK) in the surrounding cells, which accelerates cell proliferation, promotes the expression of the *Bcl-6* gene, inhibits transcription, and then inhibits apoptosis. It also promotes the expression of defective recombinase-activated genes 1 and 2 (*RAG1/2*) and enhances cellular immunity. This process also regulates energy metabolism processes, mainly glucose metabolism, and regulates the glucose-6-phosphatase analog gene (*G6pc*) to regulate glycogen metabolism and glucose homeostasis under heat stress.

In terms of protein digestion and absorption, heat stress can reduce nutrient delivery to the small intestine by causing blood to flow from internal organs to surface tissues [39]. Limitations in nutrient availability can alter intestinal functions in animals, including disrupted epithelial tight junctions, increased intestinal permeability, and reduced intestinal absorption of amino acids [40], and intestinal injury further triggers local or systemic inflammation [41]. Heat stress can also alter tissue-specific requirements for amino acids, protein synthesis and systemic protein degradation and the use of amino acids by metabolic tissues [42]. Our study showed that heat stress significantly altered protein and amino acid metabolism in MODE-K cells to resist heat stress. Glutamate, glutamine, arginine, and leucine improve the inflammatory state of the gut by preventing villous atrophy, enhancing tight junction protein synthesis, and improving intestinal barrier function. Increased levels of 3-methylhistidine and creatinine are biomarkers of increased systemic protein breakdown due to heat stress in animals and supply amino acids to maintain cellular functions [43]. Specific essential amino acids (e.g., leucine) can regulate cellular functions by activating or inhibiting signaling pathways (e.g., mammalian target protein of rapamycin, mTOR) that control nutrient sensing signaling pathways for protein synthesis [44,45]. Sammad et al. [46] also found protein digestion and absorption as an important biological pathway in granulosa cell heat stress. AMPK was one of the key signaling pathways. L-tyrosine was one of the key metabolic markers. Compared with the present study, the key regulatory genes were different. However, the functions of the genes were related to cell cycle, transmembrane transport proteins, immunity and metabolic regulation. Sammad et al. [46] investigated the biological mechanisms of the metabolome of granulosa cells in acute heat stress and found that choline, citric acid, 3-hydroxy-3-methylglutaric acid, glutamine and glycosaminoglycosides, galactosaminoglycosides, AICAR, ciliary mycolic acid, 16-hydroxyhexadecanoic acid, lysine, succinate, uridine, xanthine, and uronic acid were important metabolites in acute heat stress, involved in important metabolic pathways, including glycerophospholipid metabolism, the citric acid cycle (TCA cycle), glyoxylate and dicarboxylate metabolism, and serine, threonine, and tyrosine metabolism [47]. Clearly, findings in different studies may vary due to intrinsic cellular functions and regulation in organisms, as well as the cell type, heat stress temperature and duration used in the experimental setup, etc. The present study provides a foundation for the mining of key genes and regulatory mechanisms of heat stress in intestinal cells.

There are few studies on purine metabolism and antifolate resistance in heat stress. The differential metabolites identified in a mass spectrometry-based metabolomics study of yeast cells adapted to heat stress were involved in amino acid metabolism, energy metabolism, arginine metabolism, and purine and pyrimidine metabolism; short-term increases in spermidine and alginate were found to serve as important heat stress markers [48]. Chen et al. found that ubiquitin-dependent protein hydrolysis and purine metabolism were enhanced in catfish livers in response to acute heat stress. Pathway enrichment analysis revealed that PI3K/AKT/mTOR and protein processing pathways in the endoplasmic reticulum were regulated by heat stress [49]. Liu et al. conducted a multi-omics analysis of heat stress in livestock and poultry and found that purine metabolism was the most significantly enriched in differential genes and metabolites [50]. He et al. found that altered purine metabolism in response to oxidative stress is closely related to mitochondrial dysfunction associated with abnormalities in energy metabolism and transport [51]. The antifolate resistance pathway may be associated with cell growth inhibition caused by impaired intestinal absorption due to heat stress [52].

Mitochondrial membrane potential results revealed a gradual increase in the amount of apoptosis with increasing temperature. Gene functional analysis revealed that genes differentially regulated by heat stress at 39 °C and 41 °C mostly involved immune regulation and were closely related to protein expression, whereas genes differentially regulated at 43 °C were mostly related to regulation in nerve cells. This suggests that the heat-protective mechanisms of intestinal cells may differ depending on the degree of heat stress. Heat stress responses in an organism are essentially part of the complex neuroimmune regulation [53]. The present study corroborated the correlation between heat stress-induced intestinal injury and neuromodulation at the genetic level. Pathway analysis of significantly differential genes at 39 °C, 41 °C, and 43 °C revealed that the modulatory effects of heat on genes gradually increased with increasing temperature, shifting from the regulation of various types of metabolism to the regulation of cell proliferation, repair, apoptosis, and other processes associated with cellular immunity, energy, endocrinology, and metabolism. Particularly, the vitamin and cofactor pathways are pivotal pathways for multiple regulation. Vitamins and cofactors are required for enhanced hormone synthesis during heat stress, the conversion of certain amino acids to glucose in the gluconeogenic pathway, the breakdown of stored fats into acetyl coenzyme A, which enters the tricarboxylic acid cycle to produce energy by lipolysis, the promotion of hormone secretion in endocrine metabolism, and the maintenance of body functions by ensuring glucose production via gluconeogenesis [54,55].

The key gene Igf1 in the FoxO signaling pathway is the insulin-like growth factor, and reports have shown that heat stress can significantly increase the expression of *Igf1* [56]. *Plk1* is a polo-like kinase 1 and an important regulator of cell cycle progression. Kim et al. found that *Plk1* is an interacting protein of heat stress factor 1, and it plays an important role in heat stress-induced *HSF1* nuclear translocation [57]. In this study, both gene levels were upregulated, and the results were similar to those reported. *Ccnb2* is a member of the Cyclin family and a carcinogenic gene for various cancers. It plays an important role in regulating cell migration, proliferation, and the cell cycle [58], but there have been no reports on heat stress-related research. The three key genes involved in heat stress regulation in protein digestion and absorption pathways are all glial protein-related genes and belong to extracellular matrix proteins. *Col3a1*, known as the type Ⅲ collagen α-1 chain, plays a role in the integrity of intestinal structure and cell adhesion, migration, proliferation, and differentiation [59]. The type Ⅲ collagen in serum is a marker for distinguishing between Crohn’s disease and ulcerative colitis [60]. This gene can also be induced by nerve growth factors [61]. There are differences in the mRNA levels of *Col3a1* among different types of cells under hypoxic conditions. In this study, *Col3a1* was upregulated in intestinal cells under oxidative conditions. It has been found that the biological pathways involved in *COL3A1* expression regulation include transforming growth factor (TGF) β 1, Wnt/β- Catenin, p38 mitogen-activated protein kinase (MAPK) pathway, etc. [62,63,64]. *Col8a1* is collagen type Ⅷ α one strand. Research has shown that it is closely related to cell growth, apoptosis, cycle progression, and migration and is currently relatively abundant in cancer cell research [65]. In this study, it was found that the upregulation of the gene expression level promoted the apoptosis of MODE-K cells. *Col11a2* is type Ⅺ collagen protein α. The mutation of this gene is mainly related to genetic diseases such as cartilage dysplasia [66]. There is a co-expression phenomenon between heat stress factor 4 and a series of genes such as *COL11A2* in the occurrence and development of colon or rectal cancer [67]. At present, there have been no reports on the relationship between the three genes mentioned above and heat stress.

L-glutamic acid (metab_99), L-glutamate (metab_7290) adenosine monophosphate, adenosine diphosphate, and adenosine-5-monophosphate are metabolic markers of heat stress selected in this study. Glutamate is a functional amino acid that serves as the central hub for AA nitrogen exchange, promoting protein synthesis and degradation [68] and participating in cellular signal transduction, gene expression regulation, and metabolic cascade reactions [69]. Previous studies have shown that supplementing with L-glutamic acid or L-glutamine can improve intestinal morphological changes caused by heat stress [70], alleviate intestinal epithelial cell damage, and have adverse effects on serum parameters [71]. In this study, both amino acid contents were significantly upregulated, which may also be a way for the self-regulation of cellular heat stress. Adenosine is a neurotransmitter involved in energy metabolism and consumption. It is an important participant in the energy process. Under heat stress, cellular catabolism is enhanced, while synthetic metabolism is weakened, resulting in the consumption of a large amount of energy. In addition, He et al. found that chronic heat stress disrupts intestinal digestive enzyme activity by increasing cell apoptosis and reducing cell proliferation, causing damage to intestinal epithelial cells, which is related to the activation of adenosine 5′-monophosphate [72]. In this study, the levels of three types of adenosine were significantly upregulated, indicating that heat stress can activate energy metabolism in MODE-K cells, and the activation of adenosine may be correlated with cell damage.

In summary, this study confirms that different levels of heat stress may have different effects on cell damage and regulatory mechanisms. However, after reaching a certain temperature, the mechanisms of heat stress were similar, but the degree of regulation could be different. The five metabolic markers and six key genes identified in this study could be recognized as heat stress markers or key regulatory genes.

Integrated analysis of metabolomics and transcriptomics, the key genes, pathways, and metabolites of intestinal cell injury induced by different heat stress temperatures, was examined using metabolomics and transcriptomics association analysis, and the mechanism of cell heat stress injury was preliminarily analyzed. In addition, it was found that the pathways and metabolites were the same, but the regulatory genes were different at 41 °C and above, indicating that the same damage law may exist in different temperature ranges. Therefore, the research on the heat stress injury mechanism of 38 °C and 40 °C should be continued. At the same time, it is necessary to further confirm the key targets of heat stress-induced intestinal cell injury using Western blot and other methods. Based on this, it is highly possible to develop bioactive components or diets that can regulate and alleviate heat stress injury in intestinal cells, which will be one of the key topics in future research.

## 4. Materials and Methods

### Cells, Instruments, and Materials

The following were purchased for this study: Mouse intestinal epithelial MODE-K cells were purchased from Bei Na Biotechnology Co. (Beijing, China). Micropipettes (Sartorius, Göttingen, Germany). Clean bench (SW-CJ-1FD; Layte, Nantong, China). CO_2_ cell incubator (Thermo Fisher Scientific, WML, Waltham, MA, USA). Inverted microscope (MF52-N; Guangzhou Mshot Optoelectronic Technology Co., Guangzhou, China). Low-speed centrifuge (L3-5K; Ke-Cheng Technology Co., Taiwan, China). Water bath (HH-2; Changzhou Aohua Instrument Co., Changzhou, China). Enzyme marker (BioTek ELx800; Turner BioSystems, Sunnyvale, CA, USA). Flow cytometer (CytoFLEX; Beckman, Brea, CA, USA). Ultraperformance liquid chromatography tandem Fourier-transformed mass spectrometer (UHPLC-Q Exactive HF-X; Thermo Fisher Scientific, Guangzhou, China). Electronic balance (NewClassic MF MS105DU; Mettler, Zurich, Switzerland). PCR cycler (GeneAmp^®^9700; ABI, Benton Harbor, MI, USA). Sequencer (NovaSeq 6000; Illumina, San Diego, CA, USA). Electrophoresis power supply (DYY-6C; Beijing Liuyi Instrument Factory, Beijing, China).Penicillin-streptomycin solution (100×), 0.25% trypsin solution (containing EDTA, dissolved in PBS), Dulbecco’s modified Eagle’s medium (Wuhan Procell Life Sciences Co., Wuhan, China). Anti-β-actin antibody (Beijing Boao Sen Biotechnology Co., Beijing, China). Anti-Hsp70 antibody (3A3) (Abcam, Cambridge, UK). Horseradish peroxidase-labelled IgG (Biosharp, Chongqing, China). Foetal bovine serum (ExCell Bio, Suzhou, China). Thiazolyl blue (MedChemExpress, Shanghai, China). Dual antibody (Thermo Fisher Scientific, Guangzhou, China). Library building kits (NEXTFLEX Rapid DNA-Seq; Bio Scientific, Austin, TX, USA). Sequencing kits (NovaSeq Reagent; Illumina, San Diego, CA, USA). Pipettes (N13462C; Eppendorf, Hamburg, Germany). Cell culture dishes, 96-well cell culture plates, and cell culture flasks (Corning, New York, NY, USA).

Cell culture and passage. Culture medium: DMEM + 10% FBS + 1% (penicillin-streptomycin solution). Cell resuscitation: MODE-K cells were quickly removed from liquid nitrogen and placed in a water bath at 37 °C with gentle shaking; after dissolution of the cryopreservation agent, cells were transferred to a centrifuge tube containing 5 mL of medium and centrifuged at 1000 rpm for 5 min at room temperature. The supernatant was discarded, and the cells were harvested and suspended in complete medium containing 10% fetal bovine serum, mixed gently, and placed for culture at 37 °C, 5% CO_2_, and saturated humidity. The cells were passaged when the cell density reached 80%, and the cells were washed with PBS after the medium was discarded; then, the cells were digested with 1–2 mL of 0.25% trypsin for 1–2 min. When the cells separated from each other and rounded, the trypsin was discarded, and the complete medium was added. Single-cell suspensions were obtained by gentle shaking, passaged at a ratio of 1:3, and incubated at 37 °C, 5% CO_2_, and saturated humidity.

Cellular heat stress model induction. The MODE-K cells were grown to approximately 80% density and then incubated either at 37 °C (CON) or subjected to heat stress at 39 °C (39 °C HS), 41 °C (41 °C HS), and 43 °C (43 °C HS) for 6 h [73], followed by recovery at 37 °C for 6 h. In each group, three cell samples were used in parallel. The cells were collected for metabolomic and transcriptomic analyses.

Tight junction protein, inflammatory factors, survival/apoptotic factor mRNA expression analysis and mitochondrial membrane potential assay were conducted.

Determination of mitochondrial membrane potential and antioxidant index. Cells under different heat stress temperatures were collected when they were cultured to the experimental state. They were then measured according to MDA, LDH, GSH-PX, T-AOC, SOD, and mitochondrial membrane potential assay kits. Changes in relative mRNA expression of HSP27, HSP70, HSP90, Claudin-1, ZO-1, TNF-α, IL-1β, Bcl-2 and Bax genes were monitored. The Trizol method was used for RNA extraction, as described below: 1 mL Trizol reagent was added to the cells for mixing and transferred to a 1.5 mL RNAse-free EP tube for lysis for 10 min. Then, 200 μL of chloroform was added, mixed vigorously inverted several times, and left for 5 min at room temperature. The mixture was centrifuged for 15 min (4 °C, 12,000 rpm); the upper aqueous phase (approximately 400 μL) was transferred to a new 1.5 mL EP tube, mixed thoroughly by adding 400 μL isopropanol, and left for 10 min at room temperature. The mixture was then centrifuged at 12,000 rpm at 4 °C for 10 min, and a white RNA precipitate was visible at the bottom of the tube. The supernatant was discarded, vortexed with 1 mL of RNAse-free 75% ethanol, and centrifuged at 10,000 rpm for 5 min at 4 °C. This was repeated only once. The supernatant was discarded, and the RNA precipitate was dried in air for 5–10 min and dissolved in 20 μL of DEPC water. Then, 2 μL of dissolved RNA was used to measure the OD260, OD280 and OD260/OD280 values using a microspectrophotometer to calculate the purity and concentration of RNA. The RNA mass was estimated according to the OD260/OD280 ratio, which was between 1.8 and 2.0, to meet experimental requirements. The concentration of total RNA was calculated from the absorbance values according to the following formula:

Total RNA concentration (μg/μL) = OD260 × 40 × 10^−3^

The total RNA was stored at −80 °C for further analysis. The primer sequences used for genetic testing are listed in Table 6.

HSP70 electrophoresis. Total protein was extracted from 10^7^ cells. The proteins were separated by electrophoresis on denaturing polyacrylamide gels and blotted onto polyvinylidene difluoride membranes. The membranes were incubated with Tris-buffered saline (TBS) containing 5% skimmed milk powder at room temperature for 2 h and then probed with diluted primary antibodies (mouse anti-human β-actin and HSP70 antibodies) at 4 °C overnight. The following day, after three washes with 0.5% Tween-20/TBS at room temperature, the membranes were incubated with the secondary antibody (horseradish peroxidase-labelled goat anti-mouse IgG antibody) for 2 h. The membranes were washed three times with 0.5% Tween-20 in TBS at room temperature, and specific bands were detected using an ECL chemiluminescence kit (Shanghai Bangjing Industrial Co., Ltd., Shanghai, China).

Metabolomic analysis. Metabolomic analysis was performed using the UHPLC-Q Exactive HF-X. Sample Preparation: 10^7^ cells were added to a 2 mL centrifuge tube and a 6 mm diameter grinding bead was added. Further, 400 μL of extraction solution (methanol:water = 4:1 (*v*:*v*)) containing 0.02 mg/mL of internal standard (L-2-chlorophenylalanine) was used for metabolite extraction. Cells were ground using the Wonbio-96c frozen tissue grinder for 6 min (−10 °C, 50 Hz), followed by low-temperature ultrasonic extraction for 30 min (5 °C, 40 kHz). The cells were left at −20 °C for 30 min, centrifuged for 15 min (4 °C, 13,000 × *g*), and the supernatant was transferred to the injection vial for LC-MS/MS analysis. In addition, 20 µL of supernatant was removed from each sample and mixed as a quality control sample. LC−MS Analysis: Chromatographic conditions: ACQUITYUPLCHSST3 (100 mm × 2.1 mm i.d., 1.8 µm; Waters, Milford, CT, USA); mobile phase A consisted of 95% water + 5% acetonitrile (containing 0.1% formic acid), and mobile phase B consisted of 47.5% acetonitrile + 47.5% isopropanol + 5% water (containing 0.1% formic acid). The injection volume was 3 μL, and the column temperature was 40 °C, The elution gradient of the mobile phase is shown in Table 7. Ms conditions: Samples were ionized by electrospray, and MS signals were collected by positive and negative ion scanning modes, respectively. Scantype 70–1050 *m*/*z*, Sheathgasflowrate50 arb, Auxgasflowrate13 arb, Heatertemp 425 °C, Capillary temp 325 °C, Spray voltage(+) 3500 V, Spray voltage(−) −3500 V, S-Lens RF Level 50, Normalized collision energy 20, 40, 60 eV, Resolution 60,000 Full MS, Resolution 7500 MS2.

Raw mass spectrometry data were filtered for missing values, which were simulated (missing value recoding). Missing value removal is performed by first counting the percentage of actual missing values within the group and filtering out if it is greater than the set missing value threshold. Metabolites with more than 20% missing values within each group are removed; i.e., metabolites with more than 80% non-zero values in at least one group are retained. Filtered data will still have missing values, which are populated in this study using the minimum value of expression for that metabolite in all samples except 0. Standardization: The data were summed and normalized, QC verified RSD ≤ 30%, and Log value method was Log10 value. Quality control: The quality control (QC) samples were prepared by mixing the extract of all samples in the same volume. Each QC volume was the same as the sample, and the QC samples were processed and tested in the same way as the analytical samples. In the process of instrumental analysis, one QC sample was inserted in every five analytical samples to investigate the stability of the whole detection process.

Identification of metabolites. The raw data were imported into ProgenesisQI (Waters Corporation, Milford, CT, USA) for baseline filtering, peak identification, integration, retention time correction, peak alignment, etc. Finally, the data matrix containing retention time, mass-to-charge ratio and peak intensity information was obtained. The software used for metabolite identification was ProgenesisQIv3.0 (Waters Corporation, Milford, CT, USA), and the main databases were the mainstream public databases such as http://www.hmdb.ca/ (accessed on 25 December 2024), https://metlin.scripps.edu/ (accessed on 25 December 2024) as well as self-built databases. Search parameters: signal-to-noise ratio S/N ≥ 3. When the S/N ratio of the measurement result is higher than this threshold, the result is considered to be reliable. Molecular formula prediction was performed by using information such as the mass-to-charge ratio (*m*/*z*) of the parent ion in the primary mass spectrum, possible addition ions and isotopic peaks, and possible metabolites were identified based on a mass deviation of 10 ppm and matching with substances in the database. The secondary spectra of possible substances in the database were matched based on the corresponding daughter ions of each parent ion, scored using improved weighted mass cosine similarity, and those with a secondary fragmentation match score of 35 or more were retained for subsequent analysis. Then, the data were normalized, quality controlled, and converted. The data were compared with the KEGG and HMDB databases to obtain metabolite annotation information. Then, the data were subjected to multivariate statistical analyses, including PCA and OPLS-DA, using the ROPLS package in R. To screen for differential metabolites, we used univariate statistical analysis (*t*-tests) combined with multivariate statistical analysis (OPLS-DA/PLS-DA). Differential metabolites were screened out based on *p* < 0.05 and VIP > 1 (and |log_2_(fold change)| ≥ 1, but not by default). Results were tested using one-way ANOVA with FDR correction, applying the Student’s *t*-test for outcome testing with appropriate FDR correction. SciPy in Python (Python 3.13) was used for differential metabolite metabolic pathway enrichment analysis and VIP value analysis. Based on metabolite comparison to KEGG compound IDs, metabolic pathway information was obtained and hierarchical clustering analysis (based on the metabolite expression information in different samples, the distance of metabolites or samples was calculated, and then the metabolites or samples were classified using an iterative approach) was performed. VIP value analysis of the enriched metabolic pathways was performed using the ropls in R package(Version1.6.2, accessed on 25 December 2024).

Cellular transcriptomics analysis. Total RNA was extracted from cells (QIAzolLysis Reagent; Qiagen, New York, NY, USA), and RNA concentration and purity were determined using a Nanodrop2000 (Thermo Fisher Scientif, MA, USA). RNA integrity was assessed by agarose gel electrophoresis, and RIN values were determined using an Agilent 2100 instrument. Single builds require total RNA ≥ 1 μg, concentration ≥ 35 ng/μL, OD260/280 ≥ 1.8, and OD260/230 ≥ 1.0. mRNA was enriched using oligo (dT) magnetic beads and digested in fragmentation buffer to obtain approximately 300 bp fragments, which were isolated by magnetic bead screening. The fragments were subjected to reverse cDNA synthesis using reverse transcriptase and six-base random primers (random hexamers). cDNA sticky ends were filled using End Repair Mix, followed by the addition of an A base at the 3 end. The libraries were enriched by PCR amplification in 15 cycles. The PCR products were subjected to 2% agarose gel for the recovery of target bands. We used TBS380 (PicoGreen, Eugene, OR, USA) for quantification. Clusters were generated by bridge PCR amplification on cBot, followed by sequencing on a NovaSeq 6000 platform (read length 2 × 150 bp). The reads were matched with the GRCm39 reference genome (http://asia.ensembl.org/Mus_musculus/Info/Index, accessed on 25 December 2024) for each sample separately.

Expression analysis: Gene expression levels were calculated by the number of sequences (clean reads) localized to genomic regions (reads counts). The expression levels of genes and transcripts were quantified separately using the software RSEM (http://deweylab.github.io/RSEM/, accessed on 20 December 2024) for subsequent analysis of differential expression of genes/transcripts among different samples.

Differential expression analysis: After obtaining the ReadCounts of genes, multi-sample (≥2) projects were analyzed for differential expression of genes between samples to identify the differentially expressed genes between samples and then to study the functions of the differentially expressed genes. The software used for differential expression is DESeq2 (http://bioconductor.org/packages/stats/bioc/DESeq2/, accessed on 25 December 2024), and the default screening criteria for significantly differentially expressed genes are: FDR < 0.05&|log_2_FC| ≥ 1. When a gene satisfies these two conditions at the same time, the gene is regarded as a Differentially expressed gene (DEG).

Differential gene function annotation: Using the GO database (http://geneontology.org/, accessed on 25 December 2024), we can perform GO annotation on differentially expressed genes. Using KEGG database (https://www.genome.jp/kegg/, accessed on 25 December 2024), genes can be categorized according to the pathways they participate in or the functions they perform, and KEGG annotation of differentially expressed genes can be realized.

Enrichment of differentially expressed genes: Enrichment analysis was performed using the software Goatools (https://github.com/tanghaibao/GOatools, accessed on 25 December 2024) using Fisher’s exact test. The *p*-values were corrected for four multiple tests (Bonferroni, Holm, Sidak and false discovery rate) to control for the calculated false-positive rate, and, in general, significant enrichment was considered to exist for this GO function when the corrected *p*-value (p_fdr) was <0.05. KEGG pathway enrichment analysis was performed using the Pythonscipy software package (https://scipy.org/install/, accessed on 25 December 2024) and calculated using Fisher’s exact test. To control the calculation of false-positive rate, multiple tests were performed using the BH (FDR) method, and the corrected *p*-value was used as the threshold value of 0.05; KEGG pathways meeting this condition were defined as KEGG pathways significantly enriched in differentially expressed genes. The data were analyzed on the online platform of Majorbio Cloud Platform (www.majorbio.com, Visit from October to December 2024).

## 5. Conclusions

This study employed multi-omics association analysis to preliminarily investigate the damage mechanisms of heat stress on intestinal cells. It was found that the damage mechanisms of intestinal cells varied under different heat stress intensities (39 °C, 41 °C, and 43 °C). Increasing heat stress temperatures exacerbated the decline in cellular antioxidant capacity, upregulated HSP70 expression, enhanced inflammatory responses, disrupted plasma membrane integrity, and increased cell death. At the metabolic regulation level, the FoxO signaling pathway, antifolate resistance, taste transduction, protein digestion and absorption, and purine metabolism were identified as key pathways commonly perturbed by heat stress at different levels. At the transcriptional regulation level, with increasing heat stress temperature, the regulatory scope expanded from cellular energy metabolism, coenzyme and vitamin metabolism, and immune system to genetic replication repair, as well as cellular processes, such as transport, catabolism, growth, and death. Integrated omics analysis revealed that the FoxO signaling pathway and protein digestion/absorption pathway served as critical regulatory hubs for heat stress-induced intestinal cell damage. L-glutamic acid, L-glutamine, adenosine monophosphate (AMP), adenosine diphosphate (ADP), and 5′-adenosine monophosphate (5′-AMP) were identified as key metabolic markers, while *Col11a2*, *Col3a1*, *Col8a1*, *Igf1*, *Plk1*, and *Ccnb2* were validated as key regulatory genes involved in heat stress damage.

## Figures and Tables

**Table 1:** Heat stress-related metabolic markers in MODE-K cells.

Number	Metabolite Name	Regulate	M/Z	Rt	VIP	*p*-Value
1	2-Methylbutyroylcarnitine	up	246.17	2.6092	1.95	0.0068
2	L-Glutamic acid	up	148.06	0.5694	1.56	0.0047
3	PC(16:0/0:0)	up	518.32	6.6226	1.30	0.0412
4	5-Hydroxy-L-tryptophan	up	221.09	1.7740	2.07	0.0283
5	Phe Gly	down	223.11	2.0233	2.59	0.0060
6	Butyryl-L-carnitine	up	232.15	1.9609	1.42	0.0181
7	Alanyltryptophan	up	314.09	1.7115	1.28	0.0251
8	5′-Guanylic Acid	up	364.06	1.5317	1.68	0.0002
9	Adenosine 3′,5′-Diphosphate	up	428.04	1.3052	1.76	0.0117
10	Adenosine 3′-monophosphate	up	348.07	1.0545	1.66	0.0045
11	Imidazole Lactic Acid	up	157.06	0.6005	1.55	0.0150
12	Creatine	up	132.08	0.5926	1.48	0.0029
13	L-Carnitine	up	162.11	0.5694	1.14	0.0027
14	Guanosine 3′-monophosphate	up	364.06	1.1098	1.61	0.0019
15	Inosine 2′-phosphate	up	371.04	1.1020	3.24	0.0263
16	Phosphocreatine	up	212.04	0.5771	1.30	0.0321
17	Homomethionine	up	164.07	1.8053	2.22	0.0163
18	6-Carboxy-5,6,7,8-tetrahydropterin	up	194.07	2.0545	1.97	0.0389
19	Valylhydroxyproline	down	195.11	2.2505	1.24	0.0379
20	Adenosine monophosphate	up	348.07	0.6005	1.64	0.0086
21	NADP+	up	742.07	1.0470	1.69	0.0027
22	Indolelactic acid	up	204.07	4.7050	1.66	0.0043
23	ADP	up	426.02	0.9187	1.50	0.0154
24	8-Oxo-dGMP	up	362.05	1.1031	1.40	0.0029
25	XMP	up	363.03	1.1991	2.12	0.0193
26	3-Phenyllactic Acid	up	165.06	4.4981	1.60	0.0111
27	FAD	up	784.15	3.1929	1.63	0.0419
28	S-Adenosyl-L-homocysteine	up	383.11	1.8473	1.88	0.0031
29	GDP-Beta-L-Fucose	up	588.07	1.0951	2.08	0.0152
30	Adenosine 5′-Monophosphate	up	346.06	1.0470	1.43	0.0127
31	Guanidylic acid	up	362.05	0.5985	1.54	0.0036
32	3′-Adenylic Acid	up	346.06	0.5985	1.53	0.0132
33	Taurine	up	124.01	0.5504	1.54	0.0019
34	Fructosamine	up	214.05	0.5504	1.40	0.0148
35	L-Glutamate	up	146.05	0.5662	1.56	0.0043

Note: The VIP value of this metabolite in the OPLS-DA model between two groups; the significance test result of the difference between the two samples for this metabolite; default filtering criteria: VIP_Pred_OPLS-DA > 1, *p* < 0.05; M/Z: Mass to charge ratio, which refers to the ratio of the mass of a charged ion to its charge; Rt: retention time of charged ions in chromatography.

**Table 2:** Key pathways regulated by different levels of heat stress.

	First Category	Second Category	Pathway Description	Number of Differential Metabolites
1	Organismal Systems	Signal transduction	FoxO signaling pathway	4
2	Human Diseases	Drug resistance: antineoplastic	Antifolate resistance	6
3	Organismal Systems	Sensory system	Taste transduction	10
4	Organismal Systems	Digestive system	Protein digestion and absorption	13
5	Metabolism	Nucleotide metabolism	Purine metabolism	21

**Table 3:** Differential metabolite expression patterns in the protein digestion and absorption pathway in MODE-K cells under heat stress at 39 °C.

Metabolite Name	Gene Number	KEGG ID	M/Z	Difference Multiple	Expression Situation
L-Glutamic acid	metab_99	C00025	148.06	1.0278	up
L-Valine	metab_766	C00183	118.09	1.0146	up
L-Proline	metab_1874	C00148	116.07	1.0232	up
L-Aspartic acid	metab_2511	C00049	134.04	1.0347	up
L-His	metab_2529	C00135	156.08	1.0223	up
L-Lysine	metab_2704	C00047	147.11	1.0154	up
L-Phenylalanine	metab_3736	C00079	164.07	1.0279	up
L-Tryptophan	metab_3800	C00078	203.08	1.0301	up
L-Tyrosine	metab_5750	C00082	180.07	1.0362	up
L-Threonine	metab_7175	C00188	118.05	1.0337	up
Aspartate	metab_7287	C00049	132.03	1.0263	up
L-Glutamate	metab_7290	C00025	146.05	1.0352	up

**Table 4:** Differential metabolite expression patterns in the FoxO signalling pathway in MODE-K cells under heat stress at 41 °C.

Metabolite Name	Gene Number	KEGG ID	M/Z	Difference Multiple	Expression Situation
L-Glutamic acid	metab_99	metab_99	148.06	1.0966	up
adenosine monophosphate	metab_3502	metab_3502	348.07	1.0644	up
ADP	metab_4971	metab_4971	426.02	1.0775	up
Adenosine-5′-Monophosphate	metab_5820	metab_5820	346.06	1.0582	up
L-Glutamate	metab_7290	metab_7290	146.05	1.1038	up

**Table 5:** FoxO signaling pathway 43 °C heat stress differential metabolite expression patterns.

Metabolite Name	Gene Number	KEGG ID	M/Z	Difference Multiple	Expression Situation
L-Glutamic acid	metab_99	metab_99	148.06	1.0504	up
adenosine monophosphate	metab_3502	metab_3502	348.07	1.0787	up
ADP	metab_4971	metab_4971	426.02	1.0684	up
Adenosine-5′-Monophosphate	metab_5820	metab_5820	346.06	1.0685	up
L-Glutamate	metab_7290	metab_7290	146.05	1.0474	up

**Table 6:** Primer sequence list.

Gene	Primer	Sequence (5′-3′)	PCR Products
β-actin	Forward	CACGATGGAGGGGCCGGACTCATC	240 bp
	Reverse	TAAAGACCTCTATGCCAACACAGT	
Mus IL-1b	Forward	TCAGGCAGGCAGTATCACTC	250 bp
	Reverse	AGCTCATATGGGTCCGACAG	
Mus TNF-α	Forward	CGTCAGCCGATTTGCTATCT	206 bp
	Reverse	CGGACTCCGCAAAGTCTAAG	
Mus Bax	Forward	TTTTGCTACAGGGTTTCATCCA	181 bp
	Reverse	GTGTCCACGTCAGCAATCATC	
Mus Bcl2	Forward	AGCCCACCGTAACAATCAAG	147 bp
	Reverse	CCTGTCCCTTTGTCTTCAGC	
Mus ZO-1	Forward	CCAGCAACTTTCAGACCACC	154 bp
	Reverse	TTGTGTACGGCTTTGGTGTG	
Mus HSP70	Forward	GCAGACCTTCACCACCTACT	248 bp
	Reverse	CCTTGTCGTTGGTGATGGTG	
Mus HSP90	Forward	CTCCATGATCGGGCAGTTTG	239 bp
	Reverse	TCACCACTTCCTTGACCCTC	
Mus claudin-1	Forward	GATGTGGATGGCTGTCATTG	246 bp
	Reverse	CGTGGTGTTGGGTAAGAGGT	
Mus HSP27	Forward	AGCGCTTCGGAGAAGATGT	150 bp
	Reverse	GGTCAGGAGGAGCAGGAAG	

**Table 7:** Elution gradient of mobile phase.

	Time (min)	Flow Rate (mL/min)	A (%)	B (%)
Positive ion mode	0	0.4	100	0
	3.0	0.4	80	20
	4.5	0.4	65	35
	5.0	0.4	0	100
	6.3	0.4	0	100
	6.4	0.4	100	0
	8.0	0.4	100	0
Negative ion mode	0	0.4	100	0
	1.5	0.4	95	5
	2.0	0.4	90	10
	4.5	0.4	70	30
	5.0	0.4	0	100
	6.3	0.4	0	100
	6.4	0.4	100	0
	8.0	0.4	100	0

## Data Availability

Data is contained within the article or Supplementary Material.

## Supplementary Materials

The following supporting information can be downloaded at: https://www.mdpi.com/article/10.3390/ijms26125798/s1. The main data supporting the results of this study are available within the paper and its Supplementary Materials. Gene expression raw data were uploaded to a public database linked to http://www.ncbi.nlm.nih.gov/bioproject/1213308, accessed on 17 January 2025.
