# Supplementary material for: Integrated Multi-Omics Analysis Reveals the Mechanisms of Intestinal Cell Injury Under Different Levels of Heat Stress
Source: Int J Mol Sci. 2025 Jun 17;26(12):5798. doi: 10.3390/ijms26125798 (PMC12192742; doi:10.3390/ijms26125798)
Supplement: Supplementary file 1 [file ijms-26-05798-s001.zip › Supplementary Table.pdf]

Appendix - Table

Table S1 List of differential metabolites in the 39 ℃ HS-CON group of MODE-K cells

Table S2 List of differential metabolites in the 41 ℃ HS-CON group of MODE-K cells

Table S3 List of differential metabolites in the 43 ℃ HS-CON group of MODE-K cells

Table S4 Significant pathway enrichment analysis results of the 39 ℃ HS-CON group

Table S5 Significant pathway enrichment analysis results of the 41 ℃ HS-CON group

Table S6 Significant pathway enrichment analysis results of the 43 ℃ HS-CON group

Table S7 List of Key Genes for Heat Stress Regulation at 39 ℃

Table S8 List of Key Genes for Heat Stress Regulation at 41 ℃

Table S9 List of Key Genes for Heat Stress Regulation at 43 ℃

Table S10 39 ℃ HS-CON group gene enrichment pathway information

Table S11 41 ℃ HS-CON group gene enrichment pathway information

Table S12 43 ℃ HS-CON group gene enrichment pathway information

Table S13 Enrichment statistics of gene set and metabolic set KEGG pathway in MODE-K cells at 39℃

Table S14 Enrichment statistics of gene set and metabolic set KEGG pathway in MODE-K cells at 41℃

Table S15 Enrichment statistics of gene set and metabolic set KEGG pathway in MODE-K cells at 43℃

Table S16 Correlation analysis of differential genes and metabolites under 39 ℃ heat stress

Table S17 Correlation analysis of differential genes and metabolites under 41 ℃ heat stress

Table S1 List of differential metabolites in the 39 °C HS-CON group of MODE-K cells

Number	Metabolite name	Expression	M/Z	RT	VIP	FC(HS39/CON)	P_value
1	2-Naphthylamine	up	144.08	2.4151	1.46	1.03	0.0026
2	2-Methylbutyroylcarnitine	up	246.17	2.6092	1.95	1.05	0.0068
3	Aspartame	up	295.13	2.8669	2.37	1.08	0.0127
4	Hypoglycin B	up	334.14	3.2814	3.04	1.18	0.0329
5	L-Glutamic acid	up	148.06	0.5694	1.56	1.03	0.0047
6	3-Aminoquinoline	up	145.08	3.8904	1.46	1.04	0.0439
7	L-Valine	up	118.09	0.616	1.15	1.01	0.0130
8	Enol-phenylpyruvate	up	182.08	0.6238	1.45	1.02	0.0019
9	Pantothenic Acid	up	220.12	2.0701	1.64	1.03	0.0020
10	PI(18:1(9Z)/0:0)	up	621.30	7.2244	1.45	1.03	0.0109
11	S-Adenosylhomocysteine	up	385.13	1.6646	1.84	1.04	0.0042
12	PC(16:0/0:0)	up	518.32	6.6226	1.30	1.02	0.0412
13	PE-NMe2(18:1(11Z)/15:0)	up	764.57	6.4584	1.31	1.02	0.0379
14	5-Hydroxy-L-tryptophan	up	221.09	1.774	2.07	1.08	0.0283
15	Galactosylceramide (d18:1/16:0)	up	722.55	6.4038	1.32	1.02	0.0162
16	Inosine	up	269.09	1.7584	2.43	1.08	0.0032
17	PC(O-18:0/18:2(9Z,12Z))	up	772.62	6.0525	2.71	1.09	0.0432
18	9,9-Dimethyl-1-(sulfinylamino)decane	down	463.34	6.037	1.40	0.98	0.0495
19	1,2,3,4-Tetrahydro-2-naphthylamine	up	165.14	2.3682	3.00	1.19	0.0327
20	Indoleacrylic acid	up	188.07	2.4151	1.54	1.02	0.0020
21	PC(18:0/0:0)	down	546.35	5.865	4.47	0.69	0.0408
22	Trans-Zeatin	up	261.14	2.5935	2.69	1.14	0.0287
23	P-Tolualdehyde	down	121.07	5.7559	1.08	0.98	0.0425
24	L-Proline	up	116.07	0.5926	1.49	1.02	0.0050
25	11-Meo-fes	up	303.17	4.5694	5.06	2.13	0.0453
26	3-Hydroxy-5,5,8a-Trimethyl-3,4,4a,6,7,8-Hexahydronaphthalene-2-Carboxylic Acid	up	261.14	2.7419	2.18	1.07	0.0415
27	3-Methylindole	up	132.08	2.4151	1.47	1.03	0.0106
28	1,5-Naphthalenediamine	up	159.09	2.4073	1.57	1.03	0.0011
29	5'-Methylthioadenosine	up	298.10	2.3129	1.39	1.02	0.0132
30	N-Methylisoleucine	up	146.12	2.2505	1.67	1.04	0.0036
31	Gamma-Glutamyltyrosine	up	311.12	2.0389	2.44	1.09	0.0155
32	Phe Gly	down	223.11	2.0233	2.59	0.90	0.0060
33	Butyryl-L-carnitine	up	232.15	1.9609	1.42	1.03	0.0181
34	3-Methyl-1-phenyl-1-butanone	up	163.11	1.9456	2.24	1.08	0.0071

35	G-Glu-Val	up	247.13	1.93	2.26	1.09	0.0189
36	S-Allyl-L-cysteine	up	162.06	1.7662	1.48	1.05	0.0419
37	L-Agaridoxin	up	237.09	1.7584	1.79	1.05	0.0170
38	1-Hydroxyisoquinoline	up	146.06	1.7584	1.04	1.02	0.0039
39	9H-Purine-9-ol	up	137.05	1.7584	2.07	1.06	0.0267
40	Alanyltryptophan	up	314.09	1.7115	1.28	1.03	0.0251
41	Cytosine	up	112.05	1.7036	1.46	1.04	0.0159
42	Uridine	up	245.08	1.5863	2.95	1.18	0.0027
43	5'-Guanylic Acid	up	364.06	1.5317	1.68	1.03	0.0002
44	[(2R,5R)-5-(2-Amino-6-oxo-1H-purin-9-yl)-3,4-dihydroxyoxolan-2-yl]methyl dihydrogen phosphate	up	386.05	1.5161	1.63	1.04	0.0107
45	5-Hydroxymethyl-2-furancarboxaldehyde	up	168.07	1.5004	1.06	1.02	0.0119
46	Adenosine 3',5'-Diphosphate	up	428.04	1.3052	1.76	1.04	0.0117
47	Xanthosine 5'-Monophosphate	up	365.05	1.2114	2.47	1.13	0.0123
48	Niacinamide	up	123.06	1.1177	1.44	1.02	0.0044
49	Guanine	up	152.06	1.1098	1.53	1.03	0.0010
50	Pyridoxal	up	168.07	1.102	1.20	1.02	0.0037
51	Adenosine 3'-monophosphate	up	348.07	1.0545	1.66	1.03	0.0045
52	4-Guanidinobutanoic Acid	up	146.09	0.9607	3.47	1.31	0.0056
53	Imexon	up	112.05	0.9607	1.27	1.03	0.0225
54	Uridine diphosphate glucose	up	630.07	0.7802	1.83	1.04	0.0171
55	Uridine diphosphate-N-acetylgalactosamine	up	652.05	0.7724	1.75	1.04	0.0059
56	Imidazole Lactic Acid	up	157.06	0.6005	1.55	1.04	0.0150
57	Thiazoline	up	88.02	0.6005	1.86	1.05	0.0163
58	N,N-Dimethylarginine	up	203.15	0.5926	1.44	1.03	0.0188
59	Creatine	up	132.08	0.5926	1.48	1.02	0.0029
60	N,N'-Diethylthiourea	up	133.08	0.5926	1.53	1.03	0.0040
61	Pyrroline	up	70.07	0.5926	1.49	1.03	0.0075
62	L-Carnitine	up	162.11	0.5694	1.14	1.01	0.0027
63	L-Aspartic acid	up	134.04	0.5539	1.43	1.03	0.0372
64	L-Histidine	up	156.08	0.5461	1.33	1.02	0.0009
65	Theophylline	up	219.03	0.5383	1.91	1.05	0.0011
66	L-Lysine	up	147.11	0.4756	1.09	1.02	0.0057
67	PC(O-14:0/16:1(9Z))	up	690.54	5.9823	3.56	1.19	0.0240
68	PE(18:3(6Z,9Z,12Z)/P-18:1(9Z))	up	756.55	6.8026	2.09	1.05	0.0343
69	Guanosine 3'-monophosphate	up	364.06	1.1098	1.61	1.03	0.0000
70	Inosine 2'-phosphate	up	371.04	1.102	3.24	1.20	0.0263
71	PC(O-18:1(11Z)/16:0)	up	746.60	5.8806	1.76	1.03	0.0017
72	Sphinganine	up	302.31	5.6859	1.05	1.01	0.0203
73	Cinnamic acid	up	166.09	1.9377	1.40	1.02	0.0043

74	1-Aminocyclopropane-1-carboxylic acid	up	102.06	0.5694	1.12	1.02	0.0109
75	N-Acetyllactosamine	up	406.13	0.6005	1.37	1.02	0.0060
76	Timonacic	up	134.03	0.6005	1.98	1.05	0.0085
77	4-Hydroxycrotonic acid	up	120.07	0.5694	1.39	1.02	0.0013
78	Carnosine	up	191.09	0.4599	1.67	1.04	0.0042
79	Cyclopentanol	up	104.11	0.5539	1.80	1.03	0.0069
80	Phosphocreatine	up	212.04	0.5771	1.30	1.03	0.0321
81	Dimethyl Sulfoxide	down	79.02	0.6629	2.44	0.87	0.0301
82	Isonicotinamide	up	123.06	0.7255	1.41	1.02	0.0040
83	Uracil	up	113.03	0.9842	1.17	1.02	0.0010
84	Pyroglutamic Acid	up	130.05	1.2427	1.33	1.02	0.0072
85	Uridine diphosphategalactose	up	608.09	1.3364	1.96	1.05	0.0021
86	Xanthine	up	153.04	1.5004	2.12	1.07	0.0263
87	9,19-Cyclolanost-25-ene-3,24-diol	down	465.37	6.1305	2.17	0.93	0.0053
88	3-Methoxyanthranilate	up	168.07	1.7819	1.50	1.04	0.0263
89	Homomethionine	up	164.07	1.8053	2.22	1.09	0.0163
90	Histidylglycine	up	195.09	1.8366	1.01	1.02	0.0361
91	Zidovudine	up	250.09	1.93	2.07	1.06	0.0001
92	6-Carboxy-5,6,7,8-tetrahydropterin	up	194.07	2.0545	1.97	1.08	0.0389
93	2-n-Propylthiazolidine-4-carboxylic acid	up	176.07	2.1328	3.69	1.41	0.0083
94	Valylhydroxyproline	down	195.11	2.2505	1.24	0.97	0.0379
95	S-Butylcysteine sulfoxide	up	176.07	2.2973	1.69	1.07	0.0273
96	2-Hydroxycinnamic acid	up	182.08	1.602	1.48	1.02	0.0011
97	4-aminobenzoate	up	137.05	0.6082	2.27	1.09	0.0495
98	Adenosine monophosphate	up	348.07	0.6005	1.64	1.03	0.0086
99	Glucose-uridine-C1,5'-diphosphate	up	630.07	1.2739	1.84	1.04	0.0026
100	Indole-3-Carboxaldehyde	up	146.06	2.4151	1.68	1.04	0.0009
101	Styrene	down	105.07	5.7559	1.10	0.98	0.0216
102	NADP+	up	742.07	1.047	1.69	1.05	0.0027
103	ADP Ribose	up	540.05	1.3168	1.65	1.05	0.0341
104	NAD+	up	662.10	1.3248	1.66	1.05	0.0426
105	2-Hydroxy-3-Methylbutyric Acid	up	117.05	2.2672	1.35	1.03	0.0178
106	L-Phenylalanine	up	164.07	2.2992	1.30	1.03	0.0095
107	L-Tryptophan	up	203.08	2.9783	1.44	1.03	0.0016
108	Glutamylphenylalanine	up	293.11	3.4238	2.20	1.09	0.0305
109	Gabazine	up	332.13	3.8309	2.30	1.12	0.0316
110	Indolelactic acid	up	204.07	4.705	1.66	1.05	0.0043
111	2,4-Dinitrophenol	up	183.00	5.5028	1.89	1.07	0.0081
112	LysoPG(18:1(9Z)/0:0)	up	509.29	7.0593	1.26	1.02	0.0137
113	Uridine 2'-phosphate	up	323.03	0.5985	1.24	1.02	0.0043

114	UDP-N-acetyl-D-mannosamine	up	606.07	0.7668	1.49	1.02	0.0075
115	LysoPI(18:1(9Z)/0:0)	up	597.30	7.067	1.05	1.02	0.0167
116	Cer(d18:1/24:1(15Z))	up	692.62	6.8998	2.43	1.11	0.0022
117	LysoPI(20:4(5Z,8Z,11Z,14Z)/0:0)	up	619.29	6.7704	1.34	1.03	0.0215
118	ADP	up	426.02	0.9187	1.50	1.03	0.0154
119	Uridine 5'-diphosphoglucuronic acid	up	579.03	0.9346	1.79	1.04	0.0037
120	Inosinic acid	up	347.04	1.0951	2.43	1.10	0.0035
121	(+)-Dehydrovomifoliol	up	443.25	6.0964	1.37	1.03	0.0015
122	8-Oxo-dGMP	up	362.05	1.1031	1.40	1.02	0.0029
123	XMP	up	363.04	1.1991	2.12	1.08	0.0193
124	Fenticlor	up	266.95	1.1671	1.65	1.05	0.0248
125	Uridine diphosphate-N-acetylglucosamine	up	606.07	0.5902	1.67	1.03	0.0086
126	Guanosine	up	282.08	1.8743	1.94	1.08	0.0212
127	DNOC	up	197.02	5.7164	1.33	1.04	0.0121
128	3-Phenyllactic Acid	up	165.06	4.4981	1.60	1.04	0.0111
129	FAD	up	784.15	3.1929	1.63	1.05	0.0419
130	N-Acetyl-DL-Methionine	up	190.05	3.0683	1.13	1.02	0.0023
131	Benzenebutanoic acid, alpha-(acetylamino)-2-amino-gamma-oxo-	up	271.07	2.9703	1.40	1.04	0.0083
132	Xanthosine	up	283.07	2.2512	2.26	1.10	0.0022
133	S-Adenosyl-L-homocysteine	up	383.11	1.8473	1.88	1.07	0.0031
134	L-Tyrosine	up	180.07	1.6623	1.50	1.04	0.0016
135	Oxypurinol	up	151.03	1.5053	1.99	1.07	0.0346
136	GDP-Beta-L-Fucose	up	588.07	1.0951	2.08	1.09	0.0152
137	Adenosine 5'-Monophosphate	up	346.06	1.047	1.43	1.03	0.0127
138	Fructose-6-phosphate pyruvate	up	380.99	0.9107	1.23	1.03	0.0159
139	Uridine-5'-Monophosphate	up	323.03	0.9107	1.17	1.02	0.0090
140	Lacto-N-biose I	up	418.11	0.5985	1.20	1.02	0.0124
141	Guanidylic acid (guanosine monophosphate)	up	362.05	0.5985	1.54	1.03	0.0036
142	3'-Adenylic Acid	up	346.06	0.5985	1.53	1.03	0.0132
143	Epirubicin	down	588.17	6.0964	1.25	0.97	0.0177
144	Uric Acid	up	167.02	0.6065	1.45	1.03	0.0185
145	PE(18:1(11Z)/18:1(11Z))	down	788.55	7.1148	1.44	0.97	0.0038
146	Taurine	up	124.01	0.5504	1.54	1.04	0.0020
147	Fructosamine	up	214.05	0.5504	1.40	1.03	0.0148
148	L-Threonine	up	118.05	0.5583	1.38	1.03	0.0031
149	Threonic Acid	up	135.03	0.5742	2.16	1.09	0.0041
150	Aspartic Acid	up	132.03	0.5504	1.18	1.03	0.0433
151	L-Glutamate	up	146.05	0.5662	1.56	1.04	0.0044
152	Phenylacetylglutamine	up	309.11	2.5872	2.74	1.16	0.0105

153	Sedoheptulose	up	245.04	0.5662	1.87	1.06	0.0001

Note: RT： retention time; m/z: mass to core ratio; VIP：The variable projection importance of the first principal component in the OPLS-DA model is an indicator for screening differential metabolites; FC(HS39/CON)：39 ℃ HS-CON group differential metabolite change multiple . P_value：The P-value of Student's t-test.

Table S2 List of differential metabolites in the 41 °C HS-CON group of MODE-K cells

Number	Metabolite name	Expression	M/Z	RT	VIP	FC(HS41/CON)	P_value
1	2-Methylbutyroylcarnitine	down	246.17	2.6092	2.30	0.77	0.0016
2	DL-p-Chlorophenylalanine methyl ester hydrochloride	down	214.06	3.2579	1.23	0.94	0.0001
3	4-Chloro-L-phenylalanine	down	241.07	3.2736	1.49	0.92	0.0000
4	Hypoglycin B	up	334.14	3.2814	1.96	1.24	0.0126
5	DG(10:0/0:0/20:5(6E,8Z,11Z,14Z,17Z)-OH(5))	up	285.19	3.297	1.23	1.09	0.0337
6	L-Glutamic acid	up	148.06	0.5694	1.61	1.10	0.0000
7	P-Hydroxyubenimex	up	289.16	3.8279	2.86	1.61	0.0001
8	3-Aminoquinoline	down	145.08	3.8904	1.99	0.78	0.0001
9	[(2S,3S,4S,5S,6R)-4,5-Dihydroxy-2,6-dimethyloxan-3-yl] hydrogen sulfate	down	225.04	5.2098	1.74	0.85	0.0001
10	Taisho	down	354.10	5.2722	2.11	0.77	0.0003
11	1-Hexanol	down	246.24	5.6311	1.47	0.92	0.0002
12	(9S,10S)-9,10-dihydroxyoctadecanoate	down	334.29	5.717	1.94	0.85	0.0000
13	Capsiamide	down	302.31	5.7482	1.19	0.95	0.0014
14	S-3-oxodecanoyl cysteamine	up	246.15	5.7948	2.89	1.54	0.0001
15	D-Xylono-1,5-lactone	up	297.08	5.8258	2.70	1.48	0.0000
16	DG(15:0/18:0/0:0)	down	600.56	5.8415	1.76	0.87	0.0078
17	N-Linoleoyl Leucine	down	411.36	5.865	2.45	0.73	0.0002
18	(2S)-2-Amino-6-[(3-formylpiperidin-1-yl)amino]hexanoic acid	down	532.38	5.8963	1.53	0.91	0.0021
19	6-Methoxymellein	up	209.08	6.0839	2.58	1.44	0.0000
20	Proline betaine	up	161.13	0.499	1.98	1.18	0.0000
21	PC(14:0/0:0)	down	490.29	6.2632	1.64	0.88	0.0102
22	LysoPC(16:1(9Z)/0:0)	down	516.31	6.271	1.89	0.88	0.0005
23	PG(i-12:0/a-17:0)	down	663.45	6.3803	1.43	0.93	0.0095
24	PE(16:1(9Z)/15:0)	down	708.51	6.3803	1.86	0.84	0.0102
25	LysoPC(18:1(11Z)/0:0)	down	544.34	6.5757	1.34	0.94	0.0017
26	Methionine Sulfoxide	down	166.05	0.5771	2.77	0.64	0.0000
27	Pyridoxine	up	170.08	0.6082	1.92	1.17	0.0001
28	D-gamma-Glutamyl-D-glutamic acid	up	277.10	0.921	2.19	1.26	0.0004
29	Beta-nicotinamide adenine dinucleotide	up	664.12	1.3208	1.46	1.09	0.0035
30	Amastatin	down	516.31	7.2478	1.66	0.87	0.0034
31	S-Adenosylhomocysteine	up	385.13	1.6646	1.21	1.06	0.0017
32	Oxidized Glutathione	up	307.08	1.6411	1.85	1.19	0.0294
33	PC(16:0/0:0)	down	518.32	6.6226	1.06	0.96	0.0059
34	PE-NMe2(18:1(11Z)/15:0)	down	764.57	6.4584	1.53	0.92	0.0100
35	5-Hydroxy-L-tryptophan	down	221.09	1.774	1.43	0.88	0.0111
36	Goyaglycoside c	down	680.48	6.3803	1.74	0.87	0.0070

37	Norepinephrine	up	152.07	1.7897	1.04	1.05	0.0015
38	Isoetharine	down	520.34	6.1773	1.13	0.94	0.0365
39	Ruscogenin	down	494.32	6.1539	1.51	0.92	0.0036
40	LysoPC(14:0/0:0)	down	468.31	6.1228	1.41	0.92	0.0103
41	(2E,4E)-5-[2-Methyl-2-(1,1,4,4-tetramethyl-1,2,3,4-tetrahydronaphthalene-6-yl)cyclopropyl]-3-methyl-2,4-pentadienoic acid	up	370.28	6.1228	2.53	1.38	0.0001
42	Pantetheine 4'-phosphate	up	359.10	2.0312	1.20	1.06	0.0000
43	Solasodine	up	378.32	6.0839	2.63	1.39	0.0021
44	LysoPE(18:1(11Z)/0:0)	down	502.29	6.076	1.61	0.88	0.0165
45	LysoPA(16:0/0:0)	down	452.28	5.9589	1.40	0.92	0.0124
46	1,2,3,4-Tetrahydro-2-naphthylamine	down	165.14	2.3682	2.07	0.73	0.0076
47	Pantetheine	up	301.12	2.6795	1.55	1.11	0.0000
48	Polyoxyethylene 40 monostearate	down	346.33	5.787	1.12	0.94	0.0341
49	Sphingosine	down	300.29	5.7792	1.07	0.96	0.0000
50	(2'E,4'Z,7'Z,8E)-Colnelenic acid	up	310.24	5.7559	1.24	1.08	0.0120
51	3-ketosphingosine	down	298.27	5.7559	1.33	0.93	0.0001
52	16-Hydroxyhexadecanoic acid	down	290.27	5.717	1.78	0.89	0.0000
53	N,N-Dimethyldodecylamine-N-oxide	down	230.25	5.717	1.20	0.95	0.0024
54	Palmitelaidic acid	down	272.26	5.7093	1.24	0.94	0.0000
55	MG(i-15:0/0:0/0:0)	down	334.29	5.6311	1.56	0.89	0.0004
56	Isophorone	down	139.11	5.6078	1.89	0.85	0.0000
57	Palmitoleic acid	down	272.26	5.5921	2.28	0.74	0.0002
58	Ricinoleic acid	down	316.28	5.5686	2.34	0.73	0.0002
59	(8R,9S,10S,13S,14S,17R)-16-Fluoro-17-hydroxy-10,13-dimethyl-1,2,4,5,6,7,8,9,11,12,14,15,16,17-tetradecahydrocyclopenta[a]phenanthren-3-one	up	331.20	5.553	2.35	1.36	0.0008
60	(E)-3-(2,3-Dihydroxyphenyl)-2-propenoic acid	down	163.04	5.3659	1.21	0.93	0.0035
61	11,17-Dihydroxy-6-methyl-17-(1-propynyl)androsta-1,4,6-triene-3-one	up	317.19	5.1863	2.95	1.60	0.0000
62	2-(L-Menthoxy)ethanol	down	218.21	4.9442	1.50	0.91	0.0006
63	Lumichrome	down	243.09	4.6553	2.52	0.74	0.0000
64	11-Meo-fes	up	303.17	4.5694	3.75	2.87	0.0081
65	Gamma-Glutamylglutamic acid	up	309.13	4.4291	1.52	1.11	0.0032
66	Etozolin	up	329.09	3.8279	1.30	1.09	0.0218
67	Penicillin G	up	335.11	3.3984	2.96	1.61	0.0000
68	Fusaric acid	up	180.10	3.1799	1.06	1.06	0.0052
69	S-Prenyl-L-cysteine	up	190.09	2.9534	2.64	1.54	0.0000
70	3-Hydroxy-5,5,8a-Trimethyl-3,4,4a,6,7,8-Hexahydronaphthalene-2-Carboxylic Acid	up	261.14	2.7419	1.61	1.12	0.0073
71	1-Naphthylamine	up	144.08	2.5236	1.56	1.18	0.0359

72	N-Methylisoleucine	up	146.12	2.2505	1.09	1.06	0.0037
73	Glycyl-leucine	down	189.12	2.1563	1.42	0.88	0.0080
74	L-Valine, N-(2-hydroxy-3-butenyl)-	down	229.15	2.125	1.04	0.93	0.0318
75	Gamma-Glutamyltyrosine	up	311.12	2.0389	1.31	1.09	0.0132
76	Phe Gly	down	223.11	2.0233	2.06	0.79	0.0048
77	Butyryl-L-carnitine	down	232.15	1.9609	2.93	0.63	0.0001
78	G-Glu-Val	up	247.13	1.93	1.37	1.10	0.0009
79	Phe Ser	down	253.12	1.7819	1.72	0.79	0.0170
80	Val Val	up	217.15	1.774	1.96	1.25	0.0025
81	S-Allyl-L-cysteine	up	162.06	1.7662	2.68	1.42	0.0000
82	L-Agaridoxin	down	237.09	1.7584	2.74	0.62	0.0000
83	Tyr Gly	down	239.10	1.7584	1.51	0.88	0.0002
84	1-Hydroxyisoquinoline	down	146.06	1.7584	1.10	0.94	0.0001
85	Alanyltryptophan	down	314.09	1.7115	2.98	0.60	0.0000
86	Sparfloxacin	up	437.13	1.6411	3.02	1.61	0.0001
87	Uridine	up	245.08	1.5863	1.94	1.25	0.0010
88	Inosine 5'-Phosphate	up	349.05	1.5473	2.44	1.42	0.0001
89	5'-Guanylic Acid	up	364.06	1.5317	1.02	1.04	0.0001
90	[(2R,5R)-5-(2-Amino-6-oxo-1H-purin-9-yl)-3,4-dihydroxyoxolan-2-yl]methyl dihydrogen phosphate	up	386.05	1.5161	1.00	1.05	0.0035
91	Adenosine 3',5'-Diphosphate	up	428.04	1.3052	1.35	1.09	0.0012
92	Xanthosine 5'-Monophosphate	up	365.05	1.2114	2.85	1.56	0.0000
93	Pyridoxine 5'-phosphate	up	250.05	1.1255	2.71	1.56	0.0000
94	Niacinamide	up	123.06	1.1177	1.03	1.04	0.0017
95	Pyridoxal	down	168.07	1.102	1.07	0.94	0.0012
96	Adenosine 3'-monophosphate	up	348.07	1.0545	1.35	1.06	0.0003
97	O-Acetylcarnitine	down	204.12	1.0155	2.06	0.83	0.0002
98	4-Guanidinobutanoic Acid	up	146.09	0.9607	1.48	1.20	0.0208
99	Beta-D-Glucosamine	up	162.08	0.921	1.42	1.11	0.0065
100	Imidazole Lactic Acid	down	157.06	0.6005	1.42	0.90	0.0042
101	Thiazoline	up	88.02	0.6005	1.55	1.12	0.0009
102	Creatine	down	132.08	0.5926	1.32	0.94	0.0002
103	N,N'-Diethylthiourea	down	133.08	0.5926	1.21	0.94	0.0005
104	N-Acetyl-glucosamine 1-phosphate	up	324.05	0.5771	1.13	1.08	0.0152
105	S-Adenosylmethionine	up	399.14	0.5694	1.64	1.15	0.0005
106	L-Carnitine	down	162.11	0.5694	1.81	0.88	0.0001
107	L-Aspartic acid	up	134.04	0.5539	1.26	1.08	0.0008
108	1,6-Hexanediamine	down	158.17	0.5383	1.35	0.92	0.0043
109	Spermine	down	203.22	0.5304	1.17	0.93	0.0390
110	N(6)-Methyllysine	up	183.11	0.5304	2.09	1.22	0.0000
111	Glycerol phenylbutyrate	down	531.27	5.9276	1.57	0.90	0.0064

112	Dibutyl Phthalate	up	279.16	6.0916	2.44	1.38	0.0000
113	LysoPC(18:1(9Z)/0:0)	down	522.36	6.3257	1.35	0.94	0.0005
114	Phthalic Acid	up	149.02	6.0839	2.33	1.28	0.0001
115	Stearidonic acid	up	294.24	5.8571	1.15	1.06	0.0114
116	Guanosine 3'-monophosphate	up	364.06	1.1098	1.07	1.04	0.0000
117	Inosine 2'-phosphate	up	371.04	1.102	2.71	1.43	0.0015
118	1-Aminocyclopropane-1-carboxylic acid	up	102.06	0.5694	1.25	1.07	0.0002
119	Glutamylglutamic acid	up	277.10	0.6082	2.27	1.28	0.0004
120	Timonacic	up	134.03	0.6005	1.57	1.10	0.0007
121	4-Hydroxycrotonic acid	up	120.07	0.5694	1.09	1.05	0.0069
122	1-Ethyl-3-(dimethyl-aminopropyl)-carbodiimide	down	188.18	0.5383	1.51	0.90	0.0011
123	N6,N6,N6-Trimethyl-L-lysine	down	189.16	0.5304	1.11	0.94	0.0026
124	Carnosine	up	191.09	0.4599	1.05	1.06	0.0008
125	Cyclopentanol	up	104.11	0.5539	1.39	1.07	0.0003
126	Phosphocreatine	down	212.04	0.5771	1.94	0.82	0.0000
127	L-Methionine	up	150.06	0.6238	1.15	1.06	0.0011
128	Dimethyl Sulfoxide	down	79.02	0.6629	1.71	0.79	0.0217
129	3-ketosphinganine	down	300.29	5.6546	1.10	0.95	0.0034
130	Isonicotinamide	up	123.06	0.7255	1.17	1.06	0.0007
131	Lauryldiethanolamine	down	274.27	5.7014	1.32	0.95	0.0019
132	Uridine monophosphate (UMP)	up	325.04	0.921	1.25	1.08	0.0002
133	Uracil	up	113.03	0.9842	1.14	1.05	0.0000
134	Gamma-Glutamylcysteine	up	251.07	1.0779	2.10	1.25	0.0000
135	Pyroglutamic Acid	up	130.05	1.2427	1.22	1.06	0.0004
136	Piperidine	up	86.10	1.5708	1.07	1.04	0.0012
137	3-Methoxyanthranilate	down	168.07	1.7819	1.16	0.92	0.0009
138	Homomethionine	up	164.07	1.8053	1.68	1.17	0.0014
139	Zidovudine	up	250.09	1.93	1.47	1.10	0.0000
140	5'-Deoxy-5'-fluorouridine	up	279.10	1.93	2.43	1.40	0.0000
141	6-Carboxy-5,6,7,8-tetrahydropterin	down	194.07	2.0545	1.74	0.82	0.0031
142	2-n-Propylthiazolidine-4-carboxylic acid	up	176.07	2.1328	3.52	2.18	0.0000
143	Valylhydroxyproline	down	195.11	2.2505	2.49	0.68	0.0010
144	S-Butylcysteine sulfoxide	up	176.07	2.2973	3.12	1.71	0.0000
145	Adenosine monophosphate	up	348.07	0.6005	1.33	1.06	0.0004
146	Dioctyl phthalate	up	413.27	6.0839	2.18	1.17	0.0000
147	Glucosamine	up	162.08	0.6082	1.50	1.13	0.0103
148	NAD	up	332.56	1.5473	1.47	1.10	0.0050
149	NADP+	up	742.07	1.047	1.61	1.10	0.0012
150	Griseolic acid	up	414.04	1.0631	1.86	1.15	0.0046
151	Citric Acid	up	191.02	1.1511	2.05	1.18	0.0010

152	ADP Ribose	up	540.05	1.3168	1.61	1.09	0.0031
153	NAD+	up	662.10	1.3248	1.82	1.12	0.0018
154	5'-Thymidylic Acid	up	321.05	1.8253	3.52	1.94	0.0035
155	Gabazine	up	332.13	3.8309	1.99	1.19	0.0068
156	Indolelactic acid	down	204.07	4.705	2.36	0.77	0.0000
157	(S)-2-Acetamido-3-(4-chlorophenyl)propanoic acid	down	240.04	5.4165	1.87	0.89	0.0001
158	2-(((3,5-Dichlorophenyl)carbamoyl)oxy)-2-methyl-3-butenoic acid	down	339.96	5.5362	1.65	0.91	0.0000
159	Methyl 2-propenyl disulfide	down	299.03	5.6254	3.54	0.56	0.0000
160	Apigenin	up	269.05	5.6624	1.57	1.15	0.0174
161	PA(8:0/PGD2)	up	667.31	5.6764	1.18	1.06	0.0409
162	PGH2	up	351.22	5.7164	1.13	1.05	0.0280
163	Trospectomycin	up	419.21	5.7164	1.33	1.08	0.0252
164	Nirvanol	down	239.06	5.7644	4.16	0.41	0.0000
165	Psoralidin	down	373.05	5.8599	3.25	0.65	0.0002
166	PE(16:1/0:0)	down	450.26	5.9076	1.60	0.91	0.0088
167	Pentadecanal	up	271.23	5.9782	1.22	1.06	0.0150
168	LysoPE(P-18:0/0:0)	down	464.31	6.1117	1.50	0.93	0.0082
169	N-Arachidonoyl Isoleucine	down	438.30	6.1197	2.01	0.86	0.0020
170	PE(18:2(9Z,12Z)/18:1(9Z))	up	740.52	6.4826	1.88	1.12	0.0003
171	PE(18:0/18:1(11Z))	up	766.54	6.5552	1.72	1.10	0.0055
172	Rocuronium	down	566.35	6.621	1.63	0.93	0.0004
173	PE-NMe2(18:1(9Z)/16:1(9Z))	up	742.54	6.6325	1.20	1.04	0.0131
174	PE-NMe(15:0/18:1(9Z))	up	716.52	6.6927	1.17	1.05	0.0333
175	1-Palmitoylphosphatidylcholine	down	540.33	6.7225	1.44	0.94	0.0004
176	Uridine 2'-phosphate	up	323.03	0.5985	1.09	1.04	0.0008
177	LysoPC(0:0/18:1(9Z))	down	566.35	7.1148	1.35	0.94	0.0011
178	LysoPI(18:2(9Z,12Z)/0:0)	down	595.29	7.0988	1.43	0.94	0.0013
179	ADP	up	426.02	0.9187	1.59	1.08	0.0010
180	Manglupenone	down	457.31	6.2596	1.56	0.91	0.0178
181	LysoPC(0:0/16:0)	down	540.33	6.2437	1.51	0.93	0.0035
182	(+/-)-cis-and trans-3,5-Diethyl-1,2,4-trithiolane	down	225.01	6.2066	2.14	0.82	0.0005
183	Inosinic acid	up	347.04	1.0951	2.61	1.24	0.0001
184	(+)-Dehydrovomifoliol	down	443.25	6.0964	2.42	0.78	0.0000
185	1-(9Z-Nonadecenoyl)-glycero-3-phosphoethanolamine	down	538.31	6.0805	1.91	0.89	0.0003
186	Palmitoylcarnitine	down	436.28	6.0412	1.14	0.96	0.0241
187	8-Oxo-dGMP	up	362.05	1.1031	1.08	1.03	0.0016
188	D-Glucosaminide	up	500.21	5.9706	2.00	1.18	0.0114
189	XMP	up	363.04	1.1991	2.65	1.26	0.0003
190	Fenticlor	up	266.95	1.1671	1.96	1.14	0.0001

191	9(S)-HODE	up	295.23	5.8599	1.98	1.19	0.0036
192	1,6-anhydro-N-acetyl-beta-muramate	up	295.07	5.7964	1.09	1.04	0.0024
193	2-Quinoxalinol, 3-methyl-, 2-formate	down	225.01	5.7804	3.84	0.53	0.0000
194	N-(1,3-Benzodioxol-5-ylmethyl)-2,6-dichlorobenzamide	down	357.98	5.7644	3.15	0.64	0.0000
195	Ethyl Myristate	down	255.23	5.7484	2.05	0.87	0.0000
196	Deoxyadenosine triphosphate	down	536.00	5.6174	4.34	0.41	0.0000
197	1-Ethoxymethyl-5-fluorouracil	down	225.01	5.6174	3.81	0.50	0.0000
198	Trans-3,5-Diethyl-1,2,4,-trithiolane	down	225.01	5.5362	1.81	0.88	0.0000
199	3-Phenyllactic Acid	down	165.06	4.4981	1.20	0.95	0.0067
200	Gamma-Glu-leu	up	259.13	3.2804	1.70	1.11	0.0071
201	FAD	up	784.15	3.1929	2.51	1.22	0.0002
202	N-Acetyl-DL-Methionine	up	190.05	3.0683	1.19	1.05	0.0033
203	Xanthosine	up	283.07	2.2512	2.06	1.19	0.0004
204	Albaconazole	down	476.09	2.0584	1.80	0.89	0.0099
205	S-Adenosyl-L-homocysteine	up	383.11	1.8473	1.54	1.10	0.0007
206	GDP-Beta-L-Fucose	up	588.07	1.0951	2.23	1.20	0.0000
207	Adenosine 5'-Monophosphate	up	346.06	1.047	1.46	1.06	0.0010
208	Uridine-5'-Monophosphate	up	323.03	0.9107	1.09	1.03	0.0017
209	Guanidylic acid (guanosine monophosphate)	up	362.05	0.5985	1.16	1.04	0.0026
210	3'-Adenylic Acid	up	346.06	0.5985	1.48	1.07	0.0013
211	N-Acetyl-D-Glucosamine 6-Phosphate	up	300.05	0.5742	1.40	1.08	0.0063
212	Cyanidin 3-O-alpha-L-arabinoside	down	464.10	5.6624	2.37	0.82	0.0004
213	Epirubicin	down	588.17	6.0964	2.59	0.77	0.0015
214	Uric Acid	up	167.02	0.6065	1.76	1.10	0.0001
215	Taurine	down	124.01	0.5504	2.26	0.83	0.0007
216	Fructosamine	down	214.05	0.5504	1.00	0.96	0.0098
217	L-Threonine	up	118.05	0.5583	1.29	1.07	0.0149
218	9,10-Dihydroxystearic acid	up	297.24	5.9782	2.03	1.15	0.0006
219	Aspartic Acid	up	132.03	0.5504	1.39	1.07	0.0014
220	Gallocatechin	up	611.14	1.8173	2.56	1.30	0.0064
221	L-Glutamate	up	146.05	0.5662	1.84	1.10	0.0001
222	Phenylacetylglutamine	up	309.11	2.5872	1.68	1.14	0.0195
223	Sedoheptulose	up	245.04	0.5662	1.47	1.08	0.0011

Note: RT： retention time; m/z: mass to core ratio; VIP：The variable projection importance of the first principal component in the OPLS-DA model is an indicator for screening differential metabolites; FC(HS39/CON)：41 ℃ HS-CON group differential metabolite change multiple . P_value：The P-value of Student's t-test.

Table S3 List of differential metabolites in the 43 °C HS-CON group of MODE-K cells

Number	Metabolite name	Expression	M/Z	RT	VIP	FC(HS43/CON)	P_value
1	2-Naphthylamine	down	144.08	2.4151	1.01	0.95	0.0058
2	2-Methylbutyroylcarnitine	down	246.17	2.6092	2.56	0.71	0.0000
3	Aspartame	down	295.13	2.8669	1.14	0.93	0.0386
4	DL-p-Chlorophenylalanine methyl ester hydrochloride	down	214.06	3.2579	1.26	0.93	0.0001
5	4-Chloro-L-phenylalanine	down	241.07	3.2736	1.48	0.91	0.0000
6	DG(10:0/0:0/20:5(6E,8Z,11Z,14Z,17Z)-OH(5))	down	285.19	3.297	1.49	0.87	0.0128
7	L-Glutamic acid	up	148.06	0.5694	1.12	1.05	0.0015
8	P-Hydroxyubenimex	up	289.16	3.8279	2.49	1.48	0.0001
9	3-Aminoquinoline	down	145.08	3.8904	1.97	0.78	0.0001
10	Penicilloic acid	up	353.12	4.5537	1.42	1.09	0.0012
11	[(2S,3S,4S,5S,6R)-4,5-Dihydroxy-2,6-dimethyloxan-3-yl] hydrogen sulfate	down	225.04	5.2098	1.72	0.85	0.0000
12	Taisho	down	354.10	5.2722	2.08	0.76	0.0003
13	1-Hexanol	down	246.24	5.6311	1.34	0.93	0.0009
14	(9S,10S)-9,10-dihydroxyoctadecanoate	down	334.29	5.717	1.84	0.86	0.0000
15	Capsiamide	down	302.31	5.7482	1.28	0.94	0.0001
16	S-3-oxodecanoyl cysteamine	up	246.15	5.7948	2.27	1.35	0.0005
17	13(S)-Hydroperoxylinolenic acid	up	293.21	5.818	2.30	1.43	0.0325
18	D-Xylono-1,5-lactone	up	297.08	5.8258	2.57	1.45	0.0001
19	DG(15:0/18:0/0:0)	down	600.56	5.8415	1.72	0.88	0.0018
20	N-Linoleoyl Leucine	down	411.36	5.865	2.35	0.74	0.0006
21	(2S)-2-Amino-6-[(3-formylpiperidin-1-yl)amino]hexanoic acid	down	532.38	5.8963	1.22	0.94	0.0110
22	3beta-20(29)-Lupene-3,27-diol	up	484.41	5.9432	3.47	2.29	0.0192
23	LysoPA(0:0/18:0)	down	480.31	6.0604	1.39	0.92	0.0156
24	6-Methoxymellein	up	209.08	6.0839	2.59	1.47	0.0000
25	1,2,3,4-Tetrahydronaphthalene	up	133.10	6.1305	1.27	1.11	0.0378
26	Proline betaine	up	161.13	0.499	1.58	1.12	0.0002
27	LysoPC(16:1(9Z)/0:0)	down	516.31	6.271	1.98	0.86	0.0007
28	PG(i-12:0/a-17:0)	down	663.45	6.3803	1.39	0.93	0.0015
29	PE(16:1(9Z)/15:0)	down	708.51	6.3803	1.57	0.88	0.0194
30	LysoPC(18:1(11Z)/0:0)	down	544.34	6.5757	1.66	0.90	0.0020
31	Methionine Sulfoxide	down	166.05	0.5771	2.68	0.65	0.0000
32	Pyridoxine	up	170.08	0.6082	1.38	1.10	0.0012
33	Enol-phenylpyruvate	down	182.08	0.6238	1.01	0.96	0.0054
34	D-gamma-Glutamyl-D-glutamic acid	up	277.10	0.921	1.18	1.09	0.0241
35	Hypoxanthine	up	137.05	1.2349	1.55	1.10	0.0081

36	LysoPC(20:4(8Z,11Z,14Z,17Z)/0:0)	down	544.34	7.2478	1.69	0.88	0.0061
37	Amastatin	down	516.31	7.2478	2.49	0.72	0.0001
38	PI(18:1(9Z)/0:0)	down	621.30	7.2244	1.97	0.81	0.0163
39	PC(16:0/0:0)	down	518.32	6.6226	1.48	0.92	0.0135
40	Neogitogenin	down	496.34	6.6148	1.53	0.92	0.0005
41	PE-NMe2(18:1(11Z)/15:0)	down	764.57	6.4584	1.63	0.90	0.0013
42	5-Hydroxy-L-tryptophan	down	221.09	1.774	1.92	0.78	0.0041
43	Goyaglycoside c	down	680.48	6.3803	1.75	0.84	0.0394
44	Salbutamol	down	496.34	6.3257	1.51	0.91	0.0037
45	Inosine	up	269.09	1.7584	1.01	1.06	0.0415
46	Isoetharine	down	520.34	6.1773	1.61	0.87	0.0159
47	Ruscogenin	down	494.32	6.1539	1.80	0.89	0.0012
48	5beta-Pregnane-3alpha,20alpha-diol	up	285.26	6.1305	1.57	1.14	0.0112
49	9-Tetradecenoic acid	up	191.18	6.1305	2.65	1.45	0.0006
50	Alpha-Cyperol	up	203.18	6.1305	1.58	1.16	0.0239
51	4-Octylphenol	up	189.16	6.1305	1.28	1.11	0.0467
52	4-Heptylphenol	up	175.15	6.1305	2.40	1.42	0.0005
53	(1R,4R)-Dihydrocarvone	up	153.13	6.1305	2.17	1.36	0.0015
54	LysoPC(14:0/0:0)	down	468.31	6.1228	1.79	0.88	0.0014
55	(S)-(-)-Perillyl alcohol	up	135.12	6.1228	1.99	1.22	0.0017
56	Aglepristone	down	464.31	6.1072	1.25	0.92	0.0440
57	Solasodine	up	378.32	6.0839	2.52	1.37	0.0020
58	LysoPE(18:1(11Z)/0:0)	down	502.29	6.076	1.76	0.85	0.0288
59	LysoPA(16:0/0:0)	down	452.28	5.9589	1.75	0.87	0.0030
60	1,2,3,4-Tetrahydro-2-naphthylamine	down	165.14	2.3682	2.04	0.72	0.0073
61	Alpha-Terpineol acetate	up	214.18	5.9432	1.43	1.12	0.0226
62	Nuatigenin	down	494.32	5.8415	1.62	0.89	0.0111
63	Trans-Zeatin	down	261.14	2.5935	1.42	0.86	0.0323
64	Pantetheine	up	301.12	2.6795	1.02	1.06	0.0154
65	Sphingosine	down	300.29	5.7792	1.56	0.91	0.0000
66	Tuberculostearic acid	up	316.32	5.7637	1.82	1.12	0.0008
67	(2'E,4'Z,7'Z,8E)-Colnelenic acid	up	310.24	5.7559	1.33	1.09	0.0181
68	3-ketosphingosine	down	298.27	5.7559	1.59	0.89	0.0001
69	16-Hydroxyhexadecanoic acid	down	290.27	5.717	1.67	0.90	0.0000
70	Palmitelaidic acid	down	272.26	5.7093	1.27	0.93	0.0013
71	MG(16:0/0:0/0:0)	down	348.31	5.6859	1.23	0.92	0.0013
72	Ultram	up	296.22	5.6781	1.23	1.09	0.0037
73	(R)-Sulcatol	down	298.27	5.639	1.02	0.94	0.0170
74	MG(i-15:0/0:0/0:0)	down	334.29	5.6311	1.45	0.90	0.0000
75	Isophorone	down	139.11	5.6078	1.91	0.84	0.0000
76	Palmitoleic acid	down	272.26	5.5921	2.34	0.72	0.0000

77	Ricinoleic acid	down	316.28	5.5686	2.40	0.71	0.0000
78	(8R,9S,10S,13S,14S,17R)-16-Fluoro-17-hydroxy-10,13-dimethyl-1,2,4,5,6,7,8,9,11,12,14,15,16,17-tetradecahydrocyclopenta[a]phenanthren-3-one	up	331.20	5.553	1.95	1.26	0.0027
79	(E)-3-(2,3-Dihydroxyphenyl)-2-propenoic acid	down	163.04	5.3659	1.14	0.93	0.0034
80	11,17-Dihydroxy-6-methyl-17-(1-propynyl)androsta-1,4,6-triene-3-one	up	317.19	5.1863	2.66	1.51	0.0000
81	2-(L-Menthoxy)ethanol	down	218.21	4.9442	1.45	0.91	0.0018
82	Phenolsulfonphthalein	up	355.06	4.7882	1.58	1.12	0.0006
83	L-Proline	down	116.07	0.5926	1.19	0.95	0.0038
84	Lumichrome	down	243.09	4.6553	2.42	0.75	0.0000
85	11-Meo-fes	up	303.17	4.5694	3.46	2.68	0.0118
86	Gamma-Glutamylglutamic acid	up	309.13	4.4291	2.04	1.20	0.0001
87	Madlongiside C	down	650.40	4.3587	2.27	0.71	0.0461
88	Penicillin G	up	335.11	3.3984	3.12	1.71	0.0000
89	Fusaric acid	up	180.10	3.1799	1.43	1.11	0.0006
90	S-Prenyl-L-cysteine	up	190.09	2.9534	1.41	1.17	0.0026
91	1,5-Naphthalenediamine	down	159.09	2.4073	1.07	0.94	0.0129
92	5'-Methylthioadenosine	up	298.10	2.3129	1.62	1.10	0.0001
93	Pro Ile	down	229.15	2.2817	1.55	0.89	0.0002
94	Vomifoliol	down	247.13	2.2271	1.39	0.90	0.0173
95	Glycyl-leucine	down	189.12	2.1563	1.54	0.86	0.0025
96	L-Valine, N-(2-hydroxy-3-butenyl)-	down	229.15	2.125	1.99	0.78	0.0000
97	Phe Gly	down	223.11	2.0233	2.38	0.72	0.0001
98	Butyryl-L-carnitine	down	232.15	1.9609	3.05	0.58	0.0000
99	3-Methyl-1-phenyl-1-butanone	down	163.11	1.9456	1.18	0.91	0.0320
100	Phe Ser	down	253.12	1.7819	1.73	0.78	0.0137
101	Pro-Pro-Pro	down	310.18	1.774	1.26	0.94	0.0010
102	S-Allyl-L-cysteine	up	162.06	1.7662	2.01	1.25	0.0000
103	L-Agaridoxin	down	237.09	1.7584	2.69	0.62	0.0000
104	Tyr Gly	down	239.10	1.7584	2.30	0.71	0.0010
105	1-Hydroxyisoquinoline	down	146.06	1.7584	1.04	0.94	0.0007
106	9H-Purine-9-ol	up	137.05	1.7584	1.39	1.09	0.0051
107	Alanyltryptophan	down	314.09	1.7115	1.86	0.83	0.0009
108	Cytosine	down	112.05	1.7036	1.14	0.93	0.0014
109	Inosine 5'-Phosphate	up	349.05	1.5473	2.39	1.42	0.0000
110	5'-Guanylic Acid	up	364.06	1.5317	1.01	1.05	0.0082
111	[(2R,5R)-5-(2-Amino-6-oxo-1H-purin-9-yl)-3,4-dihydroxyoxolan-2-yl]methyl dihydrogen phosphate	up	386.05	1.5161	1.03	1.06	0.0139
112	Adenosine 3',5'-Diphosphate	up	428.04	1.3052	1.20	1.07	0.0038
113	Pyridoxine 5'-phosphate	up	250.05	1.1255	2.42	1.46	0.0000

114	Adenosine 3'-monophosphate	up	348.07	1.0545	1.42	1.07	0.0012
115	O-Acetylcarnitine	down	204.12	1.0155	2.56	0.72	0.0001
116	Imexon	down	112.05	0.9607	1.05	0.94	0.0007
117	Imidazole Lactic Acid	down	157.06	0.6005	2.05	0.79	0.0000
118	N,N-Dimethylarginine	down	203.15	0.5926	1.59	0.89	0.0008
119	Creatine	down	132.08	0.5926	1.89	0.88	0.0000
120	Pyrroline	down	70.07	0.5926	1.15	0.93	0.0082
121	N-Acetyl-glucosamine 1-phosphate	up	324.05	0.5771	1.78	1.19	0.0004
122	S-Adenosylmethionine	up	399.14	0.5694	2.47	1.34	0.0000
123	L-Carnitine	down	162.11	0.5694	2.43	0.78	0.0000
124	Theophylline	down	219.03	0.5383	1.57	0.88	0.0023
125	1,6-Hexanediamine	down	158.17	0.5383	1.04	0.95	0.0170
126	N(6)-Methyllysine	up	183.11	0.5304	1.87	1.18	0.0000
127	L-Lysine	down	147.11	0.4756	1.02	0.95	0.0017
128	Glycerol phenylbutyrate	down	531.27	5.9276	1.39	0.91	0.0499
129	Dibutyl Phthalate	up	279.16	6.0916	2.52	1.43	0.0000
130	LysoPC(18:1(9Z)/0:0)	down	522.36	6.3257	1.72	0.90	0.0001
131	6,10-Dimethyl-5(E),9-undecadien-2-one	up	177.16	6.1305	2.52	1.42	0.0013
132	Phthalic Acid	up	149.02	6.0839	2.34	1.30	0.0001
133	Pregeijerene	up	163.15	6.0525	1.09	1.06	0.0059
134	Stearidonic acid	up	294.24	5.8571	1.31	1.08	0.0197
135	Guanosine 3'-monophosphate	up	364.06	1.1098	1.08	1.04	0.0018
136	Inosine 2'-phosphate	up	371.04	1.102	2.60	1.41	0.0016
137	PC(18:1/0:0)	down	522.36	5.8258	1.30	0.93	0.0454
138	Sphinganine	down	302.31	5.6859	1.13	0.95	0.0003
139	Cinnamic acid	down	166.09	1.9377	1.03	0.96	0.0078
140	Glutamylglutamic acid	up	277.10	0.6082	1.30	1.11	0.0149
141	1-Ethyl-3-(dimethyl-aminopropyl)-carbodiimide	down	188.18	0.5383	2.77	0.66	0.0000
142	N6,N6,N6-Trimethyl-L-lysine	down	189.16	0.5304	1.74	0.85	0.0001
143	Cyclopentanol	up	104.11	0.5539	1.52	1.08	0.0001
144	Phosphocreatine	down	212.04	0.5771	1.43	0.90	0.0007
145	3-ketosphinganine	down	300.29	5.6546	1.37	0.93	0.0001
146	Lauryldiethanolamine	down	274.27	5.7014	1.24	0.95	0.0040
147	Xanthine	up	153.04	1.5004	1.41	1.10	0.0059
148	9,19-Cyclolanost-25-ene-3,24-diol	up	465.37	6.1305	3.46	1.56	0.0000
149	Homomethionine	up	164.07	1.8053	1.15	1.09	0.0240
150	LysoPC(15:0/0:0)	down	482.32	6.1773	1.15	0.94	0.0320
151	Asp Ile Glu	up	376.17	2.0155	1.40	1.15	0.0330
152	6-Carboxy-5,6,7,8-tetrahydropterin	down	194.07	2.0545	1.61	0.83	0.0188
153	2-n-Propylthiazolidine-4-carboxylic acid	up	176.07	2.1328	2.91	1.85	0.0001

154	Valylhydroxyproline	down	195.11	2.2505	2.61	0.64	0.0000
155	S-Butylcysteine sulfoxide	up	176.07	2.2973	2.49	1.48	0.0000
156	4-aminobenzoate	up	137.05	0.6082	1.58	1.14	0.0096
157	9,10-Epoxyoctadecanoic acid	up	279.23	5.9041	1.09	1.06	0.0451
158	Adenosine monophosphate	up	348.07	0.6005	1.43	1.08	0.0009
159	Dioctyl phthalate	up	413.27	6.0839	2.25	1.19	0.0000
160	Salicylic Acid	up	139.04	6.0839	1.21	1.08	0.0001
161	NADP+	up	742.07	1.047	1.26	1.06	0.0317
162	Griseolic acid	up	414.04	1.0631	1.93	1.15	0.0064
163	Citric Acid	up	191.02	1.1511	1.94	1.15	0.0024
164	5'-Thymidylic Acid	up	321.05	1.8253	3.51	1.88	0.0045
165	2-Hydroxy-3-Methylbutyric Acid	down	117.05	2.2672	2.94	0.72	0.0003
166	L-Phenylalanine	down	164.07	2.2992	1.30	0.94	0.0074
167	Adenine	up	134.05	2.9463	1.87	1.13	0.0003
168	L-Tryptophan	down	203.08	2.9783	1.15	0.96	0.0244
169	Indolelactic acid	down	204.07	4.705	2.98	0.65	0.0002
170	(S)-2-Acetamido-3-(4-chlorophenyl)propanoic acid	down	240.04	5.4165	1.48	0.94	0.0024
171	2-(((3,5-Dichlorophenyl)carbamoyl)oxy)-2-methyl-3-butenoic acid	down	339.96	5.5362	1.71	0.91	0.0000
172	Methyl 2-propenyl disulfide	down	299.03	5.6254	3.69	0.56	0.0000
173	Apigenin	up	269.05	5.6624	1.97	1.20	0.0004
174	Nirvanol	down	239.06	5.7644	4.42	0.37	0.0000
175	Psoralidin	down	373.05	5.8599	3.48	0.63	0.0000
176	PE(16:1/0:0)	down	450.26	5.9076	2.29	0.84	0.0013
177	PE(16:0/0:0)	down	452.28	5.9859	1.98	0.88	0.0028
178	LysoPE(P-18:1(9Z)/0:0)	down	462.30	6.0334	1.11	0.96	0.0495
179	LysoPE(P-18:0/0:0)	down	464.31	6.1117	2.24	0.85	0.0020
180	N-Arachidonoyl Isoleucine	down	438.30	6.1197	3.18	0.69	0.0001
181	PE(18:2(9Z,12Z)/18:1(9Z))	up	740.52	6.4826	1.77	1.10	0.0024
182	4-(undecan-5-yl)benzene-1-Sulfonic Acid	down	311.17	6.521	1.05	0.97	0.0088
183	PE(18:0/18:1(11Z))	up	766.54	6.5552	1.69	1.09	0.0114
184	Rocuronium	down	566.35	6.621	2.24	0.87	0.0001
185	1-Palmitoylphosphatidylcholine	down	540.33	6.7225	2.40	0.84	0.0005
186	Cochliobolin A	down	859.53	7.0453	1.36	0.94	0.0465
187	Dodecyl Hydrogen Sulfate	down	265.15	7.067	1.72	0.88	0.0032
188	LysoPC(0:0/18:1(9Z))	down	566.35	7.1148	1.90	0.89	0.0017
189	PS(18:1(9Z)/0:0)	down	522.28	7.1148	1.33	0.94	0.0107
190	LysoPI(16:0/0:0)	down	571.29	7.1068	1.70	0.90	0.0125
191	Hydroxy Ritonavir	down	757.28	7.0988	1.99	0.85	0.0259
192	LysoPI(18:2(9Z,12Z)/0:0)	down	595.29	7.0988	2.91	0.76	0.0004

193	LysoPI(18:1(9Z)/0:0)	down	597.30	7.067	2.05	0.89	0.0002
194	4-Decan-4-Ylbenzenesulfonic Acid	down	297.15	7.067	1.91	0.86	0.0088
195	Cer(d18:1/24:1(15Z))	up	692.62	6.8998	2.58	1.26	0.0006
196	LysoPI(20:4(5Z,8Z,11Z,14Z)/0:0)	down	619.29	6.7704	2.11	0.87	0.0042
197	PS(15:0/22:2(13Z,16Z))	down	800.55	6.5706	1.35	0.95	0.0307
198	ADP	up	426.02	0.9187	1.53	1.07	0.0025
199	Manglupenone	down	457.31	6.2596	1.67	0.89	0.0361
200	LysoPC(0:0/16:0)	down	540.33	6.2437	2.49	0.80	0.0155
201	(+/-)-cis-and trans-3,5-Diethyl-1,2,4-trithiolane	down	225.01	6.2066	2.21	0.82	0.0005
202	Inosinic acid	up	347.04	1.0951	2.60	1.22	0.0001
203	PE(18:0/0:0)	down	480.31	6.0964	1.21	0.95	0.0245
204	(+)-Dehydrovomifoliol	down	443.25	6.0964	2.56	0.78	0.0000
205	1-(9Z-Nonadecenoyl)-glycero-3-phosphoethanolamine	down	538.31	6.0805	2.70	0.80	0.0001
206	Palmitoylcarnitine	down	436.28	6.0412	1.86	0.90	0.0031
207	PE(18:1(9Z)/0:0)	down	478.29	5.9859	1.54	0.94	0.0017
208	8-Oxo-dGMP	up	362.05	1.1031	1.14	1.04	0.0087
209	D-Glucosaminide	up	500.21	5.9706	2.17	1.19	0.0089
210	9(S)-HODE	up	295.23	5.8599	2.36	1.25	0.0011
211	1,6-anhydro-N-acetyl-beta-muramate	up	295.07	5.7964	1.03	1.04	0.0188
212	2-Quinoxalinol, 3-methyl-, 2-formate	down	225.01	5.7804	3.79	0.57	0.0001
213	N-(1,3-Benzodioxol-5-ylmethyl)-2,6-dichlorobenzamide	down	357.98	5.7644	3.26	0.64	0.0000
214	Ethyl Myristate	down	255.23	5.7484	2.11	0.87	0.0000
215	Guanosine	down	282.08	1.8743	2.00	0.83	0.0124
216	PGA2	down	333.21	5.7164	1.09	0.96	0.0124
217	Deoxyadenosine triphosphate	down	536.00	5.6174	4.49	0.41	0.0000
218	1-Ethoxymethyl-5-fluorouracil	down	225.01	5.6174	3.80	0.54	0.0000
219	Trans-3,5-Diethyl-1,2,4,-trithiolane	down	225.01	5.5362	1.87	0.88	0.0000
220	3-Phenyllactic Acid	down	165.06	4.4981	1.59	0.92	0.0010
221	FAD	up	784.15	3.1929	2.40	1.19	0.0005
222	N-Acetyl-DL-Methionine	down	190.05	3.0683	1.23	0.94	0.0105
223	Benzenebutanoic acid, alpha-(acetylamino)-2-amino-gamma-oxo-	down	271.07	2.9703	1.28	0.94	0.0101
224	Ethyl beta-D-fructofuranoside	down	461.19	2.4512	1.41	0.93	0.0108
225	Albaconazole	down	476.09	2.0584	2.30	0.84	0.0009
226	3-Hydroxy-3-methylglutarate	up	161.04	1.8603	1.00	1.04	0.0146
227	S-Adenosyl-L-homocysteine	up	383.11	1.8473	1.21	1.06	0.0160
228	Oxypurinol	up	151.03	1.5053	1.72	1.10	0.0077
229	GDP-Beta-L-Fucose	up	588.07	1.0951	2.58	1.25	0.0000
230	Adenosine 5'-Monophosphate	up	346.06	1.047	1.62	1.07	0.0027
231	Fructose-6-phosphate pyruvate	down	380.99	0.9107	1.14	0.95	0.0396
232	Lacto-N-biose I	down	418.11	0.5985	1.46	0.93	0.0070

233	Guanidylic acid (guanosine monophosphate)	up	362.05	0.5985	1.18	1.04	0.0119
234	3'-Adenylic Acid	up	346.06	0.5985	1.62	1.07	0.0028
235	N-Acetyl-D-Glucosamine 6-Phosphate	up	300.05	0.5742	2.13	1.17	0.0001
236	Cyanidin 3-O-alpha-L-arabinoside	down	464.10	5.6624	2.16	0.85	0.0063
237	Epirubicin	down	588.17	6.0964	2.69	0.77	0.0010
238	Taurine	down	124.01	0.5504	3.18	0.70	0.0001
239	Fructosamine	down	214.05	0.5504	1.82	0.89	0.0024
240	3-Phosphoglycerate	down	184.99	0.5583	1.01	0.97	0.0108
241	9,10-Dihydroxystearic acid	up	297.24	5.9782	1.49	1.08	0.0085
242	L-Glutamate	up	146.05	0.5662	1.25	1.05	0.0059
243	Malic Acid	down	133.01	0.6065	1.20	0.95	0.0430

Note: RT： retention time; m/z: mass to core ratio; VIP：The variable projection importance of the first principal component in the OPLS-DA model is an indicator for screening differential metabolites; FC(HS43/CON)：43 ℃ HS-CON group differential metabolite change multiple . P_value： The P-value of Student's t-test.

Table S4 Significant pathway enrichment analysis results of the 39 °C HS-CON group

	First Category	Second Category	Pvalue_ uncorrected	Pvalue_ corrected	Quantity of key metabolites
1	Environmental Information Processing	Signal transduction	0	0.0007	5
2	Organismal Systems	Sensory system	0	0.0002	8
3	Human Diseases	Cancer: overview	0	0	10
4	Organismal Systems	Digestive system	0	0	12
5	Genetic Information Processing	Translation	0	0	12
6	Environmental Information Processing	Membrane transport	0	0	15
7	Metabolism	Global and overview maps		0	0
8	Metabolism	Nucleotide metabolism	0	0	18
9	Metabolism	Global and overview maps		0	0
10	Organismal Systems	Digestive system	0.0001	0.0014	5
11	Metabolism	Metabolism of cofactors and vitamins	0.0001	0.0014	6
12	Metabolism	Metabolism of other amino acids		0.0001	0.0013
13	Metabolism	Metabolism of other amino acids	0.0002	0.0018	6
14	Organismal Systems	Digestive system	0.0005	0.0042	5
15	Metabolism	Amino acid metabolism	0.0012	0.0095	7
16	Metabolism	Amino acid metabolism	0.0014	0.0107	5
17	Human Diseases	Drug resistance: antineoplastic	0.0028	0.0202	4
18	Metabolism	Glycan biosynthesis and metabolism	0.0042	0.0284	3
19	Human Diseases	Cardiovascular disease		0.0043	0.0273
20	Human Diseases	Substance dependence	0.0058	0.0354	3

Table S5 Significant pathway enrichment analysis results of the 41 °C HS-CON group

	First Category	Second Category	Pvalue_ uncorrected	Pvalue_ corrected	Quantity of key metabolites
1	Human Diseases	Drug resistance: antineoplastic	0	0.0004	7
2	Metabolism	Metabolism of cofactors and vitamins	0	0.0005	7
3	Organismal Systems	Sensory system	0	0.0001	10
4	Metabolism	Global and overview maps	0	0	14
5	Metabolism	Nucleotide metabolism	0	0	16
6	Metabolism	Global and overview maps	0	0	28
7	Environmental Information Processing	Signal transduction		0.0001	0.0017
8	Metabolism	Metabolism of other amino acids	0.0001	0.0017	7
9	Organismal Systems	Nervous system		0.0004	0.008
10	Organismal Systems	Endocrine system	0.0008	0.0129	7
11	Organismal Systems	Environmental adaptation	0.0009	0.014	4
12	Cellular Processes	Cellular community - eukaryotes		0.0011	0.0151
13	Metabolism	Amino acid metabolism	0.0017	0.0218	8
14	Organismal Systems	Digestive system	0.002	0.0248	7
15	Metabolism	Amino acid metabolism	0.0021	0.0238	7
16	Organismal Systems	Immune system	0.0022	0.0239	3
17	Organismal Systems	Endocrine system	0.0022	0.0239	4
18	Metabolism	Metabolism of other amino acids	0.0032	0.0289	5
19	Human Diseases	Neurodegenerative disease		0.0032	0.0289
20	Environmental Information Processing	Signaling molecules and interaction	0.0032	0.0303	7

Table S6 Significant pathway enrichment analysis results of the 43 ℃ HS-CON group

	First Category	Second Category	Pvalue_ uncorrected	Pvalue_ corrected	Quantity of key metabolites
1	Human Diseases	Cancer: overview	0	0.0001	8
2	Organismal Systems	Sensory system	0	0	11
3	Metabolism	Global and overview maps	0	0	14
4	Metabolism	Nucleotide metabolism	0	0	16
5	Environmental Information Processing	Signal transduction	0.0001	0.0031	4
6	Environmental Information Processing	Signal transduction	0.0001	0.0018	5
7	Human Diseases	Drug resistance: antineoplastic		0.0002	0.0046
8	Metabolism	Lipid metabolism	0.0012	0.0258	4
9	Organismal Systems	Digestive system		0.0013	0.0257
10	Metabolism	Global and overview maps	0.0016	0.0278	17
11	Genetic Information Processing	Translation	0.0021	0.0332	6
12	Human Diseases	Neurodegenerative disease		0.0023	0.0329
13	Environmental Information Processing	Membrane transport	0.0028	0.0379	9
14	Metabolism	Lipid metabolism	0.0029	0.0363	13
15	Organismal Systems	Nervous system	0.003	0.035	2
16	Human Diseases	Infectious disease: viral	0.003	0.035	4
17	Human Diseases	Endocrine and metabolic disease	0.0042	0.0436	3
18	Organismal Systems	Nervous system	0.0042	0.0436	6
19	Human Diseases	Infectious disease: parasitic		0.0044	0.041
20	Human Diseases	Cardiovascular disease	0.0047	0.0411	4

Table S7 List of Key Genes for Heat Stress Regulation at 39 °C

Gene_id	Gene name	FC(HS39/CON)	Pvalue	Regulate
ENSMUSG00000024352	Spata24	3.6	9.37E-06	up
ENSMUSG00000026271	Gpr35	9.07	2.67E-02	up
ENSMUSG00000026809	Spaca9	2.69	2.30E-02	up
ENSMUSG00000028463	Car9	9.14	1.83E-19	up
ENSMUSG00000030680	Pagr1a	5.08	2.56E-02	up
ENSMUSG00000031665	Sall1	29.06	3.67E-02	up
ENSMUSG00000035237	Lcat	0.18	9.99E-04	down
ENSMUSG00000036733	Rbm42	0.48	3.18E-05	down
ENSMUSG00000042379	Esm1	3.88	3.34E-19	up
ENSMUSG00000036502	Tmem255a	0.03	1.29E-02	down
ENSMUSG00000048399	Tprg	5.79	4.85E-02	up
ENSMUSG00000049313	Sorl1	0.04	3.68E-03	down
ENSMUSG00000049233	Apoo-ps	0.11	7.66E-05	down
ENSMUSG00000050982	Apol10a	0.05	4.11E-02	down
ENSMUSG00000076609	Igkc	27.25	4.39E-02	up
ENSMUSG00000090877	Hspa1b	0.13	1.70E-02	down
ENSMUSG00000058385	H2bc8	0.35	2.48E-02	down
ENSMUSG00000069268	H2bc7	0.28	3.81E-07	down
ENSMUSG00000093769	H3c14	0.01	2.94E-05	down
ENSMUSG00000079262	Slco1a6	5.99	9.95E-04	up
ENSMUSG00000091498	Mpc1-ps	2.24	6.16E-03	up
ENSMUSG00000096438	Gapdh-ps15	4.18	3.41E-02	up
ENSMUSG00000097530	Kansl2-ps	3.44	6.38E-03	up
ENSMUSG00000098404	Mrip-ps	3.54	3.29E-03	up
ENSMUSG00000116165	Pdxp	0.01	1.23E-06	down
ENSMUSG00000053117	E330013P04Rik	3.5	1.29E-02	up
ENSMUSG00000108912	E230020D15Rik	3.3	1.34E-02	up
ENSMUSG00000112489	9230116L04Rik	0.08	1.84E-02	down
ENSMUSG00000084792	1700056N10Rik	0.19	1.97E-02	down
ENSMUSG00000050299	Gm9843	3.6	1.38E-02	up
ENSMUSG00000066647	Gm5113	0.43	3.28E-02	down
ENSMUSG00000068165	Gm10233	40.49	3.17E-02	up
ENSMUSG00000083367	Gm8806	0.22	4.90E-02	down
ENSMUSG00000084858	Gm1980	2.38	4.53E-02	up
ENSMUSG00000086364	Gm11751	30.12	1.40E-03	up
ENSMUSG00000091509	Gm17066	0.39	2.82E-03	down
ENSMUSG00000095847	Gm5451	3.23	1.78E-03	up
ENSMUSG00000099190	Gm27188	4.97	4.03E-04	up
ENSMUSG00000105102	Gm35507	4.11	2.44E-02	up
ENSMUSG00000106568	Gm42814	0.2	4.04E-03	down
ENSMUSG00000107603	Gm43921	0.19	4.09E-02	down

ENSMUSG00000107928	Gm45140	0.41	3.89E-02	down
ENSMUSG00000108436	Gm44851	2.87	3.13E-02	up
ENSMUSG00000110588	Gm45774	0.44	6.20E-03	down
ENSMUSG00000113555	Gm10095	2.95	2.96E-02	up
ENSMUSG00000114378	Gm49355	0.35	4.56E-02	down
ENSMUSG00000114898	Gm49390	10.01	3.12E-02	up
ENSMUSG00000117477	Gm50092	29.35	1.01E-02	up
ENSMUSG00000072769	Gm10419	2.34	1.58E-03	up
ENSMUSG00000072930	Gm15107	0.45	1.92E-02	down

Table S8 List of Key Genes for Heat Stress Regulation at 41 °C

Gene_id	Gene name	FC(HS41/CON)	Pvalue	Regulate
ENSMUSG00000000204	Slfn4	15.49	6.04E-07	up
ENSMUSG00000001029	Icam2	0.04	2.25E-03	down
ENSMUSG00000021750	Fam107a	0.15	7.00E-20	down
ENSMUSG00000022586	Ly6i	79.71	1.19E-05	up
ENSMUSG00000024172	St6gal2	0.24	1.95E-02	down
ENSMUSG00000024770	Lipn	0.24	2.53E-03	down
ENSMUSG00000028524	Sgip1	2.3	5.93E-03	up
ENSMUSG00000029049	Morn1	0.47	1.83E-02	down
ENSMUSG00000029352	Crybb3	0.05	9.13E-03	down
ENSMUSG00000036305	Rpl39-ps	0.32	1.13E-03	down
ENSMUSG00000040280	Ndufa4l2	0.12	9.40E-03	down
ENSMUSG00000043633	Fam221b	0.14	6.45E-03	down
ENSMUSG00000043953	Ccrl2	12.77	1.11E-06	up
ENSMUSG00000044548	Dact1	0.09	2.99E-03	down
ENSMUSG00000052271	Bhlha15	0.04	3.83E-03	down
ENSMUSG00000060183	Cxcl11	147.51	5.37E-08	up
ENSMUSG00000060397	Zfp128	0.43	6.34E-04	down
ENSMUSG00000062456	Rpl9-ps6	0.19	2.78E-33	down
ENSMUSG00000063556	Gm10132	0.38	1.21E-06	down
ENSMUSG00000066632	Pgk1-rs7	4.22	9.24E-09	up
ENSMUSG00000069188	Gm13192	0.05	2.09E-02	down
ENSMUSG00000071532	Gm10335	0.34	1.99E-07	down
ENSMUSG00000072693	Gm10401	0.15	3.28E-02	down
ENSMUSG00000073600	Prob1	0.14	1.29E-02	down
ENSMUSG00000074673	Ttll9	0.14	3.75E-02	down
ENSMUSG00000077306	Gm22469	0.12	5.86E-03	down
ENSMUSG00000082035	Rpl17-ps8	0.3	9.39E-12	down
ENSMUSG00000083152	Apc-ps1	0.03	1.54E-03	down
ENSMUSG00000087624	9230111E07Rik	0.04	1.69E-02	down
ENSMUSG00000092036	Gm2244	0.02	1.18E-05	down
ENSMUSG00000092072	Gm4540	0.03	2.84E-03	down
ENSMUSG00000095887	Gm10096	0.29	1.67E-05	down
ENSMUSG00000101122	Gm17971	0.11	4.16E-03	down
ENSMUSG00000103558	Gm38220	3.05	4.90E-02	up
ENSMUSG00000103887	Gm37008	0.15	1.71E-02	down
ENSMUSG00000105339	Gm42457	0.03	2.50E-03	down
ENSMUSG00000105796	Gm42845	22.13	2.42E-02	up
ENSMUSG00000106574	Gm2451	4.5	3.60E-04	up
ENSMUSG00000110679	Rpl10-ps5	0.02	7.70E-04	down
ENSMUSG00000112392	Gm35240	0.43	2.06E-02	down
ENSMUSG00000114905	Gm48113	47.55	4.96E-04	up

ENSMUSG00000115129	Gm48916	0.24	7.78E-03	down
ENSMUSG00000116835	Gm49594	0.35	5.53E-41	down
ENSMUSG00000116908	Gm49599	0.09	9.01E-20	down
ENSMUSG00000117905	Gm50230	0.12	1.17E-02	down
ENSMUSG00002076173	Gm55118	2.57	2.75E-02	up
ENSMUSG00000025408	Ddit3	0.45	2.32E-40	down

Table S9 List of Key Genes for Heat Stress Regulation at 43 °C

Gene_id	Gene name	FC(HS43/CON)	Pvalue	Regulate
ENSMUSG00000000386	Mx1	3.83	7.40E-04	up
ENSMUSG00000003379	Cd79a	29.70	1.56E-09	up
ENSMUSG00000003477	Inmt	0.15	3.00E-16	down
ENSMUSG00000006310	Zbtb32	3.44	6.43E-03	up
ENSMUSG00000006311	Etv2	65.86	3.11E-04	up
ENSMUSG00000006378	Gcat	2.45	1.77E-17	up
ENSMUSG00000006411	Nectin4	20.86	3.63E-03	up
ENSMUSG00000006469	Slc34a3	43.66	9.17E-03	up
ENSMUSG00000007030	Vwa7	12.37	9.18E-04	up
ENSMUSG00000009350	Mpo	11.04	4.70E-03	up
ENSMUSG00000012042	4930579F01Rik	34.69	3.05E-03	up
ENSMUSG00000014773	Dll1	33.68	1.16E-06	up
ENSMUSG00000017344	Vtn	109.53	9.85E-06	up
ENSMUSG00000017737	Mmp9	19.64	8.42E-04	up
ENSMUSG00000018341	Il12rb2	65.13	4.57E-06	up
ENSMUSG00000019647	Sema6a	324.02	1.75E-09	up
ENSMUSG00000020383	Il13	30.02	1.85E-02	up
ENSMUSG00000020609	Apob	111.25	2.26E-05	up
ENSMUSG00000020627	Klhl29	6.81	4.30E-02	up
ENSMUSG00000020912	Krt12	64.12	1.56E-11	up
ENSMUSG00000021135	Slc10a1	47.34	1.15E-05	up
ENSMUSG00000021298	Gpr132	17.89	8.44E-03	up
ENSMUSG00000021314	Amph	24.02	4.04E-02	up
ENSMUSG00000021363	Mak	5.09	7.23E-03	up
ENSMUSG00000021590	Spata9	3.64	3.62E-04	up
ENSMUSG00000021872	Rnase10	117.44	7.65E-06	up
ENSMUSG00000022454	Nell2	981.22	9.55E-12	up
ENSMUSG00000022619	Mapk8ip2	6.55	4.29E-03	up
ENSMUSG00000022803	Popdc2	9.94	4.73E-05	up
ENSMUSG00000022853	Ehhadh	82.58	3.14E-05	up
ENSMUSG00000022878	Adipoq	31.65	3.84E-03	up
ENSMUSG00000023903	Mmp25	35.10	1.19E-05	up
ENSMUSG00000023914	Mep1a	4.72	6.29E-05	up
ENSMUSG00000024114	Prss41	31.96	1.50E-02	up
ENSMUSG00000024842	Cabp4	17.52	3.94E-14	up
ENSMUSG00000025014	Dntt	149.37	7.50E-07	up
ENSMUSG00000025141	Myadml2	31.13	8.27E-04	up
ENSMUSG00000025348	Itga7	6.68	1.58E-03	up
ENSMUSG00000025408	Ddit3	0.34	7.21E-05	down
ENSMUSG00000025977	Boll	3.65	3.84E-03	up

ENSMUSG00000026173	Plcd4	4.28	4.91E-07	up
ENSMUSG00000026175	Vil1	12.83	2.21E-04	up
ENSMUSG00000026582	Sele	0.46	2.91E-03	down
ENSMUSG00000026725	Tnn	6.05	1.02E-05	up
ENSMUSG00000027360	Hdc	22.68	7.82E-06	up
ENSMUSG00000027880	Slc25a54	7.79	6.16E-03	up
ENSMUSG00000027967	Neurog2	896.85	6.52E-09	up
ENSMUSG00000028012	Rrh	2.75	3.74E-04	up
ENSMUSG00000028860	Sytl1	6.52	5.29E-05	up
ENSMUSG00000029193	Cckar	204.96	5.54E-08	up
ENSMUSG00000029368	Alb	49.64	4.87E-03	up
ENSMUSG00000029641	Rasl11a	84.62	3.80E-07	up
ENSMUSG00000029648	Flt1	5.46	5.26E-15	up
ENSMUSG00000030087	Klf15	0.23	6.88E-03	down
ENSMUSG00000030214	Plbd1	137.51	1.30E-06	up
ENSMUSG00000030278	Cidec	24.97	8.65E-07	up
ENSMUSG00000030329	Pianp	4.91	1.24E-04	up
ENSMUSG00000030468	Siglecg	0.37	2.04E-02	down
ENSMUSG00000030653	Gm45837	69.77	2.34E-04	up
ENSMUSG00000030786	Itgam	99.46	2.82E-05	up
ENSMUSG00000030895	Hpx	3.28	2.84E-02	up
ENSMUSG00000031227	Magee1	2.63	5.71E-03	up
ENSMUSG00000031326	Cdx4	57.73	8.57E-04	up
ENSMUSG00000031637	Lrp2bp	13.84	9.98E-09	up
ENSMUSG00000031932	Gpr83	179.21	2.10E-07	up
ENSMUSG00000032238	Rora	6.33	7.28E-04	up
ENSMUSG00000032297	Celf6	42.11	4.82E-04	up
ENSMUSG00000032517	Mobp	47.93	6.97E-04	up
ENSMUSG00000032719	Sbspon	3.80	4.33E-04	up
ENSMUSG00000032911	Cspg4	2.33	1.66E-02	up
ENSMUSG00000033187	BC016579	95.28	1.40E-04	up
ENSMUSG00000033368	Trim69	38.40	2.14E-18	up
ENSMUSG00000033644	Piwil2	5.04	1.32E-02	up
ENSMUSG00000034683	Ppp1r1c	115.15	2.98E-05	up
ENSMUSG00000034764	1700006J14Rik	0.02	3.12E-04	down
ENSMUSG00000035576	L3mbtl1	143.25	1.61E-06	up
ENSMUSG00000036305	Rpl39-ps	2.93	4.85E-04	up
ENSMUSG00000036452	Arhgap26	0.48	8.29E-12	down
ENSMUSG00000036655	Colec11	19.00	8.92E-04	up
ENSMUSG00000037346	Hrh4	69.87	2.01E-04	up
ENSMUSG00000037548	H2-DMb2	115.23	2.10E-05	up
ENSMUSG00000037727	Avp	39.25	3.87E-03	up
ENSMUSG00000038199	Iqca1l	49.63	5.03E-03	up
ENSMUSG00000038305	Spats2l	2.18	6.71E-03	up

ENSMUSG00000038390	Gpr162	0.38	2.68E-02	down
ENSMUSG00000038932	Tcfl5	4.40	3.34E-02	up
ENSMUSG00000038980	Rbbp8nl	25.86	3.76E-02	up
ENSMUSG00000039239	Tgfb2	13.60	1.81E-03	up
ENSMUSG00000039579	Grin3a	3.47	2.50E-02	up
ENSMUSG00000039981	Zc3h12d	35.42	2.61E-05	up
ENSMUSG00000040017	Saa4	87.34	2.18E-04	up
ENSMUSG00000040046	Tph1	3.59	3.48E-02	up
ENSMUSG00000040247	Tbc1d10c	3.13	8.94E-03	up
ENSMUSG00000040283	Btnl9	35.05	6.36E-05	up
ENSMUSG00000040367	Lrrd1	14.43	5.85E-05	up
ENSMUSG00000041073	Nacad	16.13	5.54E-08	up
ENSMUSG00000041117	Ccdc8	19.14	1.59E-05	up
ENSMUSG00000041872	Il17f	73.86	1.90E-08	up
ENSMUSG00000042250	Pglyrp4	26.26	7.77E-04	up
ENSMUSG00000042678	Myo15	3.19	9.59E-03	up
ENSMUSG00000042707	Dnali1	24.34	3.35E-07	up
ENSMUSG00000042988	Notum	43.67	8.88E-03	up
ENSMUSG00000043410	Hfm1	95.21	6.10E-04	up
ENSMUSG00000043441	Gpr149	0.13	4.84E-02	down
ENSMUSG00000043456	Zfp536	231.59	4.43E-09	up
ENSMUSG00000043670	Diras1	59.29	5.58E-08	up
ENSMUSG00000043925	Olfr544	9.54	1.83E-02	up
ENSMUSG00000044176	Spink10	4.02	7.26E-03	up
ENSMUSG00000044724	Gpr152	42.91	1.32E-06	up
ENSMUSG00000045034	Ankrd34b	148.96	3.74E-06	up
ENSMUSG00000045350	Fam186a	15.36	3.69E-04	up
ENSMUSG00000045551	Fpr1	3.41	1.95E-03	up
ENSMUSG00000046213	Cym	20.42	2.70E-02	up
ENSMUSG00000046487	Mospd4	12.58	3.08E-03	up
ENSMUSG00000047021	Cfap65	45.68	5.63E-03	up
ENSMUSG00000047384	A730013G03Rik	69.66	2.80E-04	up
ENSMUSG00000047419	Cmya5	4.89	1.48E-02	up
ENSMUSG00000047420	Fam180a	5.19	2.33E-04	up
ENSMUSG00000047953	Gp5	9.21	1.88E-03	up
ENSMUSG00000048644	Ctxn1	3.48	3.12E-03	up
ENSMUSG00000048772	Tmem53	0.26	2.27E-02	down
ENSMUSG00000048960	Prex2	5.26	1.08E-03	up
ENSMUSG00000050217	Lgsn	113.41	9.34E-06	up
ENSMUSG00000050359	Sprr1a	51.85	1.62E-03	up
ENSMUSG00000050578	Mmp13	0.33	1.02E-06	down
ENSMUSG00000050612	Txndc2	7.20	2.66E-03	up
ENSMUSG00000051243	Islr2	15.99	2.30E-06	up
ENSMUSG00000051498	Tlr6	0.40	1.34E-06	down

ENSMUSG00000051639	Fbl-ps2	3.00	2.90E-02	up
ENSMUSG00000051648	Kctd19	722.67	1.51E-09	up
ENSMUSG00000052013	Btla	107.33	2.20E-05	up
ENSMUSG00000052160	Pld4	7.81	1.44E-03	up
ENSMUSG00000052270	Fpr2	152.51	4.19E-08	up
ENSMUSG00000053687	Dpep2	0.29	8.14E-11	down
ENSMUSG00000053863	Mepe	39.91	5.31E-03	up
ENSMUSG00000054252	Fgfr3	21.22	6.19E-03	up
ENSMUSG00000054320	Lrrc36	305.74	1.37E-10	up
ENSMUSG00000054360	Bsx	119.33	8.16E-06	up
ENSMUSG00000054555	Adam12	6.57	4.14E-04	up
ENSMUSG00000054679	Srsf12	25.87	3.58E-02	up
ENSMUSG00000054892	Txk	46.01	2.33E-03	up
ENSMUSG00000055102	Zfp819	16.30	2.27E-02	up
ENSMUSG00000055216	9430025C20Rik	27.39	4.12E-10	up
ENSMUSG00000056900	Usp13	77.54	3.15E-05	up
ENSMUSG00000058254	Tspan7	15.39	4.92E-02	up
ENSMUSG00000058743	Kcnj14	75.37	1.45E-10	up
ENSMUSG00000058809	Hspd1-ps3	45.76	3.87E-32	up
ENSMUSG00000059213	Ddn	15.94	1.08E-10	up
ENSMUSG00000059562	Ccdc154	16.19	1.87E-04	up
ENSMUSG00000064225	Paqr9	137.13	4.25E-06	up
ENSMUSG00000064288	H4c12	36.16	8.71E-05	up
ENSMUSG00000064360	mt-Nd3	0.46	1.26E-09	down
ENSMUSG00000065701	Rny1	9.01	4.44E-03	up
ENSMUSG00000065947	mt-Nd4l	0.20	2.91E-08	down
ENSMUSG00000067338	Tuba3b	417.82	7.00E-12	up
ENSMUSG00000067795	4930444P10Rik	61.37	3.07E-04	up
ENSMUSG00000068349	Gml	161.23	4.64E-07	up
ENSMUSG00000068740	Celsr2	33.05	9.20E-07	up
ENSMUSG00000069816	Olfr23	29.76	4.15E-02	up
ENSMUSG00000070330	Tmem235	27.57	7.34E-03	up
ENSMUSG00000070720	Tmem200b	41.71	1.01E-02	up
ENSMUSG00000071226	Cecr2	20.85	3.14E-03	up
ENSMUSG00000073408	Mucl3	27.79	5.47E-03	up
ENSMUSG00000073414	Mpig6b	147.56	4.45E-08	up
ENSMUSG00000073600	Prob1	0.06	3.99E-07	down
ENSMUSG00000073739	Gm16287	4.76	1.36E-02	up
ENSMUSG00000073761	4933427I04Rik	8.63	4.99E-04	up
ENSMUSG00000074388	Gm5544	41.81	1.42E-03	up
ENSMUSG00000074483	Bglap	42.19	3.92E-03	up
ENSMUSG00000074657	Kif5a	9.20	4.19E-06	up
ENSMUSG00000078153	Psme2b	34.35	2.18E-02	up
ENSMUSG00000078606	Gvin2	0.09	1.65E-13	down

ENSMUSG00000079484	Phyhd1	0.10	1.79E-43	down
ENSMUSG00000080021	Gm5915	49.01	3.79E-04	up
ENSMUSG00000081272	Ap2m1-ps	5.45	1.70E-04	up
ENSMUSG00000081605	Gm15953	14.62	7.70E-07	up
ENSMUSG00000081965	Gm11620	3.53	3.27E-03	up
ENSMUSG00000082741	Gm9703	32.48	1.79E-04	up
ENSMUSG00000083899	Gm12346	2.37	3.52E-02	up
ENSMUSG00000086179	Gm14317	4.62	2.23E-07	up
ENSMUSG00000086187	Gm12860	96.40	1.73E-05	up
ENSMUSG00000086231	Rapgef4os3	41.65	2.30E-02	up
ENSMUSG00000086275	1700121C08Rik	8.85	1.37E-05	up
ENSMUSG00000086413	Gm12415	21.61	1.93E-06	up
ENSMUSG00000087141	Plcxd2	4.65	2.04E-03	up
ENSMUSG00000087223	4930442L01Rik	51.79	1.95E-03	up
ENSMUSG00000087231	E230016M11Rik	0.35	3.97E-06	down
ENSMUSG00000087445	Gm14286	0.09	1.06E-10	down
ENSMUSG00000088088	Rmrp	277.25	1.04E-159	up
ENSMUSG00000089669	Tnfsf13	0.42	3.14E-02	down
ENSMUSG00000089670	Gm16581	211.49	8.00E-09	up
ENSMUSG00000089697	Gm15947	42.03	4.09E-03	up
ENSMUSG00000089961	Gm16567	0.20	1.78E-05	down
ENSMUSG00000091709	Gm17189	37.92	6.67E-03	up
ENSMUSG00000091983	Olfr457	11.97	7.97E-03	up
ENSMUSG00000092253	H2-Q3	77.45	3.26E-04	up
ENSMUSG00000092518	Garin5b	434.13	2.55E-11	up
ENSMUSG00000093402	Gm18588	0.02	8.11E-05	down
ENSMUSG00000093445	Lrch4	0.11	2.66E-36	down
ENSMUSG00000094840	Muc3a	248.63	1.18E-08	up
ENSMUSG00000095621	Gm15085	0.00	3.24E-10	down
ENSMUSG00000097233	Gm17552	420.46	3.10E-11	up
ENSMUSG00000097333	Zfp87	0.50	1.87E-06	down
ENSMUSG00000097471	5830432E09Rik	4.37	9.31E-03	up
ENSMUSG00000097619	4833422M21Rik	10.13	2.12E-04	up
ENSMUSG00000097715	Gpr137b-ps	0.19	1.09E-05	down
ENSMUSG00000099032	Tcf24	8.54	5.69E-04	up
ENSMUSG00000099102	Gm11983	145.54	7.46E-07	up
ENSMUSG00000101791	2210011K15Rik	2.48	1.72E-02	up
ENSMUSG00000102828	Gm38182	0.45	9.10E-03	down
ENSMUSG00000102908	Gm7558	24.01	4.56E-02	up
ENSMUSG00000105851	9130604C24Rik	8.69	4.44E-04	up
ENSMUSG00000106188	Gm9710	0.28	1.40E-03	down
ENSMUSG00000108030	9530062K07Rik	9.59	6.30E-06	up
ENSMUSG00000108079	Gm44210	8.87	7.31E-06	up
ENSMUSG00000108236	0610033M10Rik	264.04	1.89E-04	up

ENSMUSG00000108368	Gm45053	3.79	1.10E-02	up
ENSMUSG00000108774	Gm45136	43.74	1.97E-02	up
ENSMUSG00000109032	Gm7972	39.70	1.27E-02	up
ENSMUSG00000109299	Gm45164	23.87	4.94E-02	up
ENSMUSG00000110080	Gm6145	9.41	1.36E-05	up
ENSMUSG00000110104	Gm45717	9.89	1.16E-03	up
ENSMUSG00000110368	Gm45518	3.59	1.70E-03	up
ENSMUSG00000110492	Gm5358	39.93	5.30E-03	up
ENSMUSG00000110619	Gm7850	164.74	2.52E-07	up
ENSMUSG00000110679	Rpl10-ps5	3.38	1.63E-02	up
ENSMUSG00000111028	Gm5922	33.10	5.24E-07	up
ENSMUSG00000111137	Gm2553	35.77	1.29E-02	up
ENSMUSG00000111683	Gm49367	56.07	9.14E-04	up
ENSMUSG00000112719	Gm45925	9.17	9.46E-03	up
ENSMUSG00000114196	Gm47547	0.43	1.08E-03	down
ENSMUSG00000114231	Gm40968	3.00	3.87E-02	up
ENSMUSG00000114245	Percc1	57.74	8.42E-04	up
ENSMUSG00000115852	Gm52969	0.04	4.59E-02	down
ENSMUSG00000116534	Gm49731	21.43	2.01E-02	up
ENSMUSG00000116831	Gm30505	35.97	9.94E-03	up
ENSMUSG00000116994	Gm49684	15.42	4.45E-02	up
ENSMUSG00000117257	Gm4948	38.02	6.37E-03	up
ENSMUSG00000117312	Gm49931	21.29	1.04E-04	up
ENSMUSG00000117525	Gm50034	135.70	1.51E-06	up
ENSMUSG00000117780	Gm3734	6.91	1.44E-05	up
ENSMUSG00000117922	Gm50397	37.84	7.73E-03	up
ENSMUSG00000117923	Gm30593	297.98	3.70E-05	up
ENSMUSG00000117966	1700061A03Rik	50.12	2.30E-04	up
ENSMUSG00000117988	Gm8663	62.25	4.40E-04	up
ENSMUSG00000118030	Gm50163	8.92	1.83E-07	up
ENSMUSG00000118094	Gm52988	39.85	5.90E-03	up
ENSMUSG00000118928	Gm25547	29.81	3.61E-02	up
ENSMUSG00000119562	n-R5s130	136.33	9.78E-08	up
ENSMUSG00000119648	Snord3b3	39.77	2.65E-02	up
ENSMUSG00000119761	Snord3b2	41.75	2.28E-02	up
ENSMUSG00000119851	Snord3b4	41.75	2.28E-02	up
ENSMUSG00000119954	Gm35546	8.44	2.31E-05	up

Table S10 39 ℃ HS-CON group gene enrichment pathway information

	Pathway id	Description	Ratio_in_study	Padjust	First Category	Second Category	Gene_names
1	mmu05410	Hypertrophic cardiomyopathy	8/252	0.0224	Human Diseases	Cardiovascular disease	Igf1;Itga2b;Tnnt2;Sgcd;Itga11;Edn1;Agt;Itgb4
2	mmu00750	Vitamin B6 metabolism	3/252	0.0370	Metabolism	Metabolism of Cofactors and Vitamins	Pdxp;Aox4;Aox3
3	mmu05414	Dilated cardiomyopathy	7/252	0.0565	Human Diseases	Cardiovascular disease	Igf1;Tnnt2;Sgcd;Itga11;Itga2b;Agt;Itgb4
4	mmu04974	Digestion and absorption of proteins	7/252	0.0866	Organismal Systems	digestive system	Cpb1;Col11a2;Col8a1;Cpa2;Col3a1;Ctrl;Col1a1
5	mmu04972	Pancreatic secretion	7/252	0.0905	Organismal Systems	digestive system	Pla2g3;Amy2a2;Cel;Amy2a3;Ctrl;Cpa2;Cpb1
6	mmu00910	Nitrogen metabolism	3/252	0.0908	Metabolism	energy metabolism	Car15;Car14;Car9
7	mmu04933	AGE-RAGE signal transduction pathway in complications of diabetes	6/252	0.1884	Human Diseases	Endocrine and metabolic diseases	Ccl2;Edn1;Col3a1;Fn1;Col1a1;Agt
8	mmu04613	The formation of neutrophil extracellular traps	8/252	0.1906	Organismal Systems	immune system	C3;Selp;Itga2b;H3c14;Hdac9;H2bc12;H2bc7;Ncf4
9	mmu00500	Metabolism of starch and sucrose	3/252	0.2776	Metabolism	Carbohydrate metabolism	Pygm;Amy2a3;Amy2a2
10	mmu04114	Oocyte meiosis	6/252	0.2799	Cellular Processes	Cell growth and death	Igf1;Ccnb2;Espl1;Plk1;Camk2a;Camk2b

Note: (1) Pathway ID: Path number; (2) Description: Specific description of KEGG pathway; (3) Ratio_ In_ Study: The proportion of KEGG annotated genes in the target gene set that fall into the KEGG pathway, where the numerator is the number of genes annotated into the KEGG pathway in the target gene set and the denominator is the total number of genes annotated with KEGG in the target gene set; (4) Adjust: The corrected P-value is corrected using the BH method by default. (5) First category: A branch of the KEGG metabolic pathway. Annotations for tables of the same type are the same.

Table S11 41 °C HS-CON group gene enrichment pathway information

	Pathway id	Description	Ratio_in_study	Padjust	First Category	Second Category	Gene_names
1	mmu03030	DNA replication	16/1410	7.2023E-09	Genetic Information Processing	Copy and Repair	Rfc2;Rpa1;Pold1;Rfc3;Mcm5;Mcm6;Mcm7;Prim2;Rfc5;Mcm4;Pola1;Dna2;Mcm3;Pole2;Lig1;Pole
2	mmu04110	cell cycle	31/1410	1.1982E-07	Cellular Processes	Cell growth and death	Bub1b;Gadd45a;Pkmyt1;Ttk;Cdc20;Mcm7;Smc3;Cdkn2c;Pttg1;Mcm3;Cdc45;Ccnb1;Rbl1;Mcm6;Plk1;Cdc25b;Bub1;E2f2;Chek1;Ccne2;Espl1;Cdc25c;Mcm4;Cdk1;Cdc6;Ccna2;Ccnb2;Cdkn1a;Mad2l1;Dbf4;Mcm5
3	mmu05164	Influenza A	36/1410	1.0921E-06	Human Diseases	Infectious diseases: viruses	Oas3;Oas1g;Ifnb1;Try4;Adar;Eif2ak2;2210010C04Rik;Pml;Irf9;Tlr3;Icam1;Cyct;Mx2;Ticam1;Stat1;Oas1a;Gm10184;Calcoco2;Fdps;Try5;Tlr7;Cxcl10;Oas1b;Ifih1;Ccl5;Xpo1;Irf7;Trim25;Ddx58;Oas2;Ccl2;Rsad2;Stat2;Tnfsf10;Il6;Prkcb
4	mmu00910	Nitrogen metabolism	8/1410	4.6825E-05	Metabolism	energy metabolism	Car8;Cps1;Car15;Car12;Car14;Car7;Car6;Car9
5	mmu03460	Fanconi anemia pathway	14/1410	0.0001	Genetic Information Processing	Copy and Repair	Eme1;Fanca;Ercc1;Fanci;Rmi2;Fancd2;Rpa1;Rad51;Rad51c;Poln;Brca1;Brip1;Usp1;Cenps
6	mmu03430	Mismatch repair	8/1410	0.0004	Genetic Information Processing	Copy and Repair	Rfc2;Exo1;Rfc5;Msh6;Rpa1;Rfc3;Lig1;Pold1
7	mmu00010	Glycolysis/ Glucose Production	15/1410	0.0005	Metabolism	Carbohydrate metabolism	Hkdc1;Pgm1;Aldh9a1;Aldoc;Pfkl;Eno1b;Eno1;Pgk1;Ldha;Acss2;Pck2;Gapdh;Pgam1;Aldh3a1;Pkm
8	mmu03440	homologous recombination	11/1410	0.0007	Genetic Information Processing	Copy and Repair	Eme1;Rpa1;Topbp1;Rad54l;Rad51;Bard1;Brca1;Brip1;Rad51c;Rad54b;Pold1
9	mmu04668	TNF signaling	21/1410	0.0010	Environmental Information	signal transduction	Ccl5;Icam1;Ccl20;Map2k6;Vcam1;G

		pathway			Processing		m5431;Ifnb1;Mmp3;Mlkl;Mapk10;Cxcl1;Map3k8;Edn1;Ifi47;Birc3;Ccl2;Il6;Cxcl10;Casp7;Il15;Jun
10	mmu04512	ECM receptor interaction	17/1410	0.0014	Environmental Information Processing	Signal molecules and interactions	Lama3;Lamc2;Cd47;Col6a2;Sdc1;Agrn;Vwf;Tnc;Npnt;Dmp1;Itga2b;Col6a1;Tnr;Thbs2;Col1a1;Col6a3;Hmmr

Table S12 43 °C HS-CON group gene enrichment pathway information

	Pathway id	Description	Ratio_in_study	Pvalue	First Category	Second Category	Gene_names
1	mmu04142	lysosome	54/3577	0.0009	Cellular Processes	Transportation and catabolism	Hexb;Atp6v0a4;Gm2a;Ap4b1;Ctns;Mcoln1;Atp6ap1;Smpd1;Arsa;Ap1g2;Psap;Ap3m1;Gnptg;Pla2g15;Cd68;Asah1;Fuca2;Naga;Ap3b2;Ctss;Arsb;Ctsa;Arsg;Acp2;Scarb2;Entpd4;Ppt2;Ctso;Lgmn;Lamp2;Ctsk;Ctsh;Ppt1;Slc17a5;Hyal2;Ctsd;Sgsh;Idua;Sumf1;Ap3s2;Dmxl2;Napsa;Hexa;Cln5;Slc11a1;Man2b1;Nagpa;Wdr7;Neu1;Glb1;Gaa;Npc1;Entpd4b;Tpp1
2	mmu04110	cell cycle	48/3577	0.0090	Cellular Processes	Cell growth and death	Bub1b;Gadd45a;Pkmyt1;Ywhah;Ttk;Anapc2;E2f2;Cdc26;Gadd45g;Mcm7;Smc3;Cdkn2d;Ccnd3;Mcm3;Anapc15;Cdc45;Rbl1;Mcm6;Plk1;Orc1;Cdc25b;Bub1;Gadd45b;Chek1;Fzr1;Wee1;Tgfb3;Bub3;Tgfb2;Mdm2;Espl1;Cdc25c;Mcm4;Prkdc;Smc1b;Cdk1;Cdc14b;Cdc6;Ccna2;Ccnb2;Cdc20;Skp1;Orc2;Cdc7;Sfn;Dbf4;Anapc10;Mcm5
3	mmu04146	Peroxisome	35/3577	0.0156	Cellular Processes	Transportation and decomposition	Acox2;Mlycd;Pmvk;Acox3;Pxmp2;Mvk;Pex11b;Baat;Pipox;Abcd1;Hmgcl;Hacl1;Pex10;Pex3;Mpv17;Pex16;Paox;Ehhadh;Pex14;Slc27a2;Hmgcll1;Xdh;Far2;Pxmp4;Pecr;Ddo;Mpv17l;Acsl1;Nos2;Abcd2;Pex11a;Dhrs4;Crot;Acaa1a;Nudt12
4	mmu03460	Fanconi anemia pathway	23/3577	0.0277	Genetic Information Processing	Copy and Repair	Slx1b;Fanca;Fancb;Rmi2;Fancd2;Hes1;Palb2;Ercc1;Rpa2;Fanci;Top3b;Faap24;Brip1;Rad51c;Brca1;Eme1;Fancc;Cenps;Cenpx;Usp1;Polh;Rad51;Ube2t
5	mmu05323	Rheumatoid arthritis	32/3577	0.0786	Human Diseases	Immune diseases	Atp6v0a4;Flt1;Atp6v1e1;Tnfsf13;Atp6ap1;Il15;Jun;Icam1;H2-DMb2;Tek;Atp6v0e;H2-DMb1;Atp6v1e2;Il23a;Fos;Il18;Tgfb3;Tnfsf13b;Tgfb2;Atp6v1c2;Il1a;Ctsk;Vegfa;Ccl5;Atp6v1g2;Il11;Atp6v1a;Ccl2;Cd28;Cxcl1;Il6;H2-Ob
6	mmu04610	Complement and coagulation cascade	33/3577	0.0854	Organismal Systems	Immune system	C4bp;Cr2;C1s1;Vwf;Cr1l;C1ra;F7;C1rb;C2;F12;Procr;F5;Cfh;Serping1;Clu;Tfpi;Kng1;Itgax;Itgam;Masp1;F8;Cd59b;Vtn;Cd46;Serpine1;C4b;C5ar1;Bdkrb2;C8g;F2;C1s2;Cfi;Serpinf2
7	mmu00280	Degradation of valine,	23/3577	0.0899	Metabolism	Amino acid metabolism	Acsf3;Hmgcs1;Mmut;Hmgcs2;Acat3;Hmgcl;Mccc1;Hadhb;Eh

		leucine, and isoleucine					hadh;Mccc2;Hmgcll1;Aacs;Acads;Aox4;4930438A08Rik;Aldh6a1;Abat;Acat2;Aldh9a1;Dbt;Dld;Bckdha;Acaa1a
8	mmu00330	Metabolism of arginine and proline	22/3577	0.0955	Metabolism	Amino acid metabolism	Ckm;Maoa;Nos2;Srm;Azin2;Aldh4a1;Sat2;Maob;Cndp2;Ckb;Ckmt2;Gamt;Cndp1;Amd1;Sat1;Carns1;Amd2;Prodh2;Got1l1;Hoga1;Aldh9a1;P4ha1
9	mmu00520	Metabolism of amino acid sugars and nucleotide sugars	20/3577	0.0966	Metabolism	Carbohydrate metabolism	Hexb;Pmm1;Gfpt2;Renbp;Cyb5r1;Fcsk;Cyb5rl;Gnpnat1;Pgm3;Gale;Nanp;Hkdc1;Hk2;Gmppa;Galk2;Hexa;Galk1;Npl;Amdhd2;Gnpda1
10	mmu00640	Metabolism of propionate	14/3577	0.0988	Metabolism	Carbohydrate metabolism	Aldh6a1;Mlycd;Acads;Mmut;Acss2;Dbt;Ldha;Dld;Ehhadh;Acss1;Bckdha;Abat;Acox3;Ldhc

Table S13 Enrichment statistics of gene set and metabolic set KEGG pathway in MODE-K cells at 39°C

	Path Description	first kind	second kind	Metabolite quantity	number of genes
1	Hypertrophic cardiomyopathy (HCM)	Human Diseases	Cardiovascular disease	0	8
2	Vitamin B6 metabolism	Metabolism	Metabolism of Cofactors and Vitamins	1	3
3	Dilated cardiomyopathy (DCM)	Human Diseases	Cardiovascular disease	0	7
4	Digestion and absorption of proteins	Organismal Systems	digestive system	12	7
5	Pancreatic secretion	Organismal Systems	digestive system	0	7
6	Nitrogen metabolism	Metabolism	energy metabolism	2	3
7	AGE-RAGE signal transduction pathway in complications of diabetes	Human Diseases	Endocrine and metabolic diseases	1	6
8	Regulation of actin cytoskeleton in cells	Cellular Processes	Cellular movement	0	9
9	The interaction between ECM and receptors	Environmental Information Processing	Signal molecules and interactions	0	5
10	Oocyte meiosis	Cellular Processes	Cell growth and death	0	6
11	Starch and sucrose metabolism	Metabolism	Carbohydrate metabolism	2	3
12	The regulation of TRP channels by inflammatory mediators	Organismal Systems	Sensory system	0	6
13	Tyrosine metabolism	Metabolism	Amino acid metabolism	1	3
14	Drug metabolism - cytochrome P450	Metabolism	Biodegradation and metabolism of alien organisms	0	4
15	PI3K Akt signaling pathway	Environmental Information Processing	signal transduction	2	11
16	Digestion and absorption of fat	Organismal Systems	digestive system	0	3
17	Arrhythmic right ventricular cardiomyopathy (ARVC)	Human Diseases	Cardiovascular disease	0	4

18	Cholinergic synapse	Organismal Systems	nervous system	0	5
19	HIF-1 signaling pathway	Environmental Information Processing	signal transduction	0	5
20	Phenylalanine metabolism	Metabolism	Amino acid metabolism	5	2
21	Cushing's syndrome	Human Diseases	Endocrine and metabolic diseases	2	6
22	Hepatitis C	Human Diseases	Infectious diseases: viral	0	6
23	Metabolism of Taurine and Low Taurine	Metabolism	Metabolism of other amino acids	3	2
24	FoxO signaling pathway	Environmental Information Processing	signal transduction	5	3
25	Carbon metabolism in cancer	Human Diseases	Cancer: Overview	10	1
26	Biosynthesis of amino acid tRNA	Genetic Information Processing	translate	12	0
27	Nucleotide metabolism	Metabolism	Global and Overview Maps	16	0

Table S14 Enrichment statistics of gene set and metabolic set KEGG pathway in MODE-K cells at 41℃

	Path Description	first kind	second kind	Metabolite quantity	number of genes
1	DNA replication	Genetic Information Processing	Copy and Repair	0	16
2	cell cycle	Cellular Processes	Cell growth and death	0	31
3	Influenza A	Human Diseases	Infectious diseases: viruses	0	36
4	Nitrogen metabolism	Metabolism	energy metabolism	2	8
5	Fanconi anemia pathway	Genetic Information Processing	Copy and Repair	0	14
6	Mismatch repair	Genetic Information Processing	Copy and Repair	0	8
7	Glycolysis/Glucose Production	Metabolism	Carbohydrate metabolism	0	15
8	homologous recombination	Genetic Information Processing	Copy and Repair	0	11
9	TNF signaling pathway	Environmental Information Processing	signal transduction	0	21
10	ECM receptor interaction	Environmental Information Processing	Signal molecules and interactions	0	17
11	NOD like receptor signaling pathway	Organismal Systems	immune system	0	31
12	Digestion and absorption of proteins	Organismal Systems	digestive system	7	19
13	Hepatitis C	Human Diseases	Infectious diseases: viruses	0	26
14	Progesterone mediated oocyte maturation	Organismal Systems	endocrine system	0	17
15	Rheumatoid arthritis	Human Diseases	Immune diseases	0	16
16	Pancreatic secretion	Organismal Systems	digestive system	1	19
17	Pyruvate metabolism	Metabolism	Carbohydrate metabolism	0	10
18	HIF-1 signaling pathway	Environmental Information Processing	signal transduction	1	19

19	measles	Human Diseases	Infectious diseases: viruses	0	23
20	galactose metabolism	Metabolism	Carbohydrate metabolism	0	8
21	α - Linolenic acid metabolism	Metabolism	Lipid metabolism	2	7
22	Oocyte meiosis	Cellular Processes	Cell growth and death	0	20
23	lysosome	Cellular Processes	Transportation and catabolism	1	21
24	Gap junction	Cellular Processes	Cell Community - Eukaryotes	4	15
25	Base excision repair	Genetic Information Processing	Copy and Repair	0	8
26	Metabolism of fructose and mannose	Metabolism	Carbohydrate metabolism	1	8
27	P53 signaling pathway	Cellular Processes	Cell growth and death	0	13
28	Nucleotide cleavage repair	Genetic Information Processing	Copy and Repair	0	9
29	Digestion and absorption of fat	Organismal Systems	digestive system	1	9
30	Glycerophospholipid metabolism	Metabolism	Lipid metabolism	11	16
31	Cell membrane DNA sensing pathway	Organismal Systems	immune system	0	11
32	β - Alanine metabolism	Metabolism	Metabolism of other amino acids	5	7
33	Herpes simplex virus 1 infection	Human Diseases	Infectious diseases: viruses	0	57
34	Ether lipid metabolism	Metabolism	Lipid metabolism	0	9
35	Phenylalanine metabolism	Metabolism	Amino acid metabolism	2	5
36	Hypertrophic cardiomyopathy (HCM)	Human Diseases	Cardiovascular disease	0	14
37	Parkinson's disease	Human Diseases	Neurodegenerative diseases	4	33
38	Toll like receptor signaling pathway	Organismal Systems	immune system	0	15
39	Metabolism of glycine, serine, and threonine	Metabolism	Amino acid metabolism	4	8
40	ABC transportation vehicles	Environmental Information Processing	Membrane transport	10	9
41	Central carbon metabolism in cancer	Human Diseases	Cancer: Overview	6	11

42	Phagosome	Cellular Processes	Transportation and catabolism	0	27
43	The biosynthesis of valine, leucine, and isoleucine	Metabolism	Amino acid metabolism	1	2
44	Metabolism of cysteine and methionine	Metabolism	Amino acid metabolism	8	9
45	Glycerol metabolism	Metabolism	Lipid metabolism	0	10
46	malaria	Human Diseases	Infectious diseases: Parasites	0	9
47	Contraction of vascular smooth muscle	Organismal Systems	circulatory system	2	19
48	FoxO signaling pathway	Environmental Information Processing	signal transduction	5	17
49	Resilience	Human Diseases	Drug resistance: anti-tumor	7	3

Table S15 Enrichment statistics of gene set and metabolic set KEGG pathway in MODE-K cells at 43°C

	Path Description	first kind	second kind	Metabolite quantity	number of genes
1	Nucleotide metabolism	Metabolism	Global and Overview Maps	14	1
2	lysosome	Cellular Processes	Transportation and catabolism	1	1
3	cell cycle	Cellular Processes	Cell growth and death	0	48
4	Peroxisome	Cellular Processes	Transportation and catabolism	0	35
5	Fanconi anemia pathway	Genetic Information Processing	Copy and Repair	0	23
6	galactose metabolism	Metabolism	Carbohydrate metabolism	0	15
7	Degradation of valine, leucine, and isoleucine	Metabolism	Amino acid metabolism	0	23
8	Rheumatoid arthritis	Human Diseases	Immune diseases	0	32
9	Metabolism of arginine and proline	Metabolism	Amino acid metabolism	6	22
10	Complement and coagulation cascade	Organismal Systems	immune system	0	33
11	Biosynthesis of Terpenoid Skeletons	Metabolism	Metabolism of Terpenes and Polyketones	0	12
12	Metabolism of Propionate Esters	Metabolism	Carbohydrate metabolism	0	14
13	Metabolism of amino acid sugars and nucleotide sugars	Metabolism	Carbohydrate metabolism	4	20
14	Metabolism of glycine, serine, and threonine	Metabolism	Amino acid metabolism	4	18
15	DNA replication	Genetic Information Processing	Copy and Repair	0	15
16	homologous recombination	Genetic Information Processing	Copy and Repair	0	17
17	β - Metabolism of alanine	Metabolism	Metabolism of other amino acids	0	14

18	Biosynthesis of mannose type O-glycans	Metabolism	The biosynthesis and metabolism of sugars	0	11
19	ABC transportation vehicles	Environmental Information Processing	Membrane transport	9	19
20	Type I diabetes	Human Diseases	Endocrine and metabolic diseases	0	12
21	Glycolipid Biosynthesis - Ganglio Series	Metabolism	Carbohydrate biosynthesis and metabolism	0	5
22	Starch and sucrose metabolism	Metabolism	Carbohydrate metabolism	0	13
23	Glycosylphosphatidylinositol (GPI) anchor biosynthesis	Metabolism	Carbohydrate biosynthesis and metabolism	3	11
24	Primary immunodeficiency	Human Diseases	Immune diseases	0	14
25	MicroRNAs in Cancer	Human Diseases	Cancer: Overview	0	48
26	Pyruvate metabolism	Metabolism	Carbohydrate metabolism	1	16
27	FoxO signaling pathway	Environmental Information Processing	signal transduction	5	40
28	Metabolism of selenium compounds	Metabolism	Metabolism of other amino acids	0	8
29	malaria	Human Diseases	Infectious diseases: Parasites	0	19
30	Glycolysis/Glucose Production	Metabolism	Carbohydrate metabolism	1	22
31	Butyrate metabolism	Metabolism	Carbohydrate metabolism	2	11
32	Inflammatory bowel disease (IBD)	Human Diseases	Immunological diseases	0	21
33	pyrimidine metabolism	Metabolism	Nucleotide metabolism	2	19
34	Adipose cytokine signaling pathways	Organismal Systems	endocrine system	2	23
35	Digestion and absorption of proteins	Organismal Systems	digestive system	6	32
36	sphingomyelin metabolism	Metabolism	Lipid metabolism	4	18
37	Metabolism of cysteine and methionine	Metabolism	Amino acid metabolism	5	18

38	Hypertrophic cardiomyopathy (HCM)	Human Diseases	Cardiovascular disease	0	28
39	Tryptophan metabolism	Metabolism	Amino acid metabolism	2	1
40	Legion disease	Human Diseases	Infectious diseases: bacteria	0	20
41	Arrhythmic right ventricular cardiomyopathy (ARVC)	Human Diseases	Cardiovascular disease	0	24
42	Gustatory conduction	Organismal Systems	Sensory system	11	17

Table S16 Correlation analysis of differential genes and metabolites under 39 ℃ heat stress

Gene Name	correlation	L-valine	L-lysine	L-Proline	l-glutamic acid	L-tryptophan	L-phenylalanine	L-tyrosine	L-glutamic acid	L-valine
ENSMUSG0000006819（Col8a1）	positive correlation	-	*	-	*	*	*	*	*	*
ENSMUSG00000001506(Col1a1)	positive correlation	*	**	**	**	**	**	*	**	**
ENSMUSG00000024330(Col11a2)	negative correlation	*	-	*	*	*	*	*	*	**

注：*，p＜0.05；** ，p＜0.01。

Table S17 Correlation analysis of differential genes and metabolites under 41 ℃ heat stress

Gene Name	correlation	L-glutamic acid	Adenosine monophosphate	ADP	Adenosine-5 '-Monophosphate	L-Glutamate
ENSMUSG00000040618 (Pck2)	positive correlation	***	***	***	***	***
ENSMUSG00000078566 （Bnip3）	positive correlation	***	***	***	***	***
ENSMUSG00000109324 (Prmt1)	positive correlation	**	***	***	***	***
ENSMUSG00000030161 (Gabarapl1)	positive correlation	***	***	***	***	***
ENSMUSG00000036390 (Gadd45a)	positive correlation	**	**	***	***	**
ENSMUSG00000023067 (Cdkn1a)	positive correlation	***	***	***	***	***

注：**，p＜0.01；*** ，p＜0.001。
