# Supplementary material for: Integrated Multi-Omics Analysis Reveals the Mechanisms of Intestinal Cell Injury Under Different Levels of Heat Stress
Source: Int J Mol Sci. 2025 Jun 17;26(12):5798. doi: 10.3390/ijms26125798 (PMC12192742; doi:10.3390/ijms26125798)
Supplement: Supplementary file 1 [file ijms-26-05798-s001.zip › Supplementary Figure.pdf]

Supplementary Figure

Figure S1. Statistical chart of KEGG Compound metabolite classification profile

Figure S2. Annotated Venn and bar plots of differential genes and differential metabolite KEGG enrichment pathways in the 39 °C HS-CON group

Figure S3. Annotated Venn and bar plots of differential genes and differential metabolite KEGG enrichment pathways in the 41 °C HS-CON group

Figure S4. Annotated Venn and bar plots of differential genes and differential metabolite KEGG enrichment pathways in the 43 °C HS-CON group

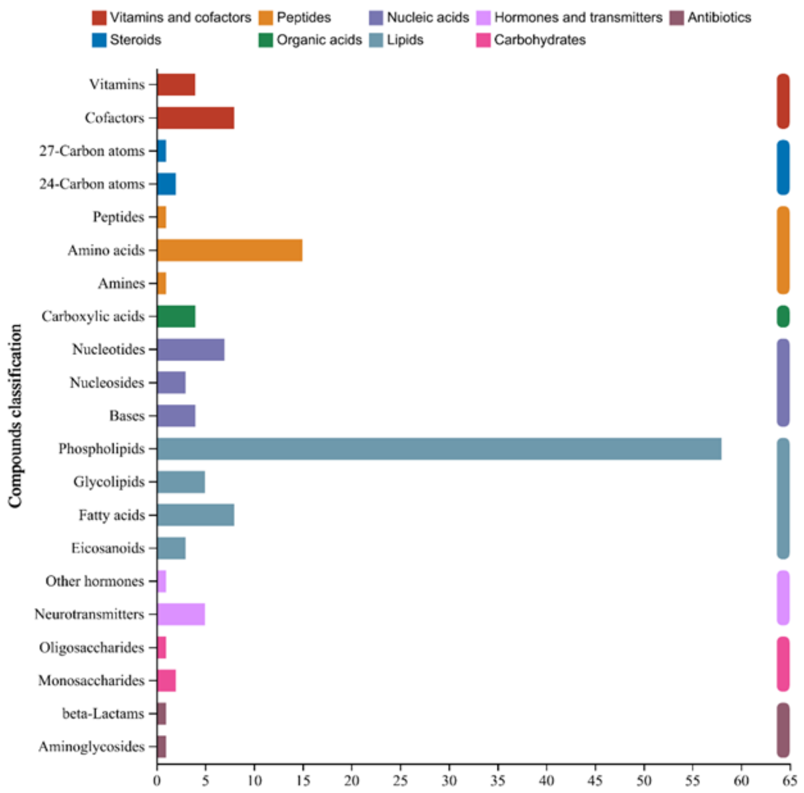

Figure S1 Statistical chart of KEGG Compound metabolite classification profile

Note: The vertical axis represents the classification of KEGG compounds, and the horizontal axis represents the number of compounds annotated to that type; The color of the bar indicates belonging to the first level classification category of compounds; The color of the bar represents different categories of metabolic pathways. The annotations for the same type of image in the following text are the same.

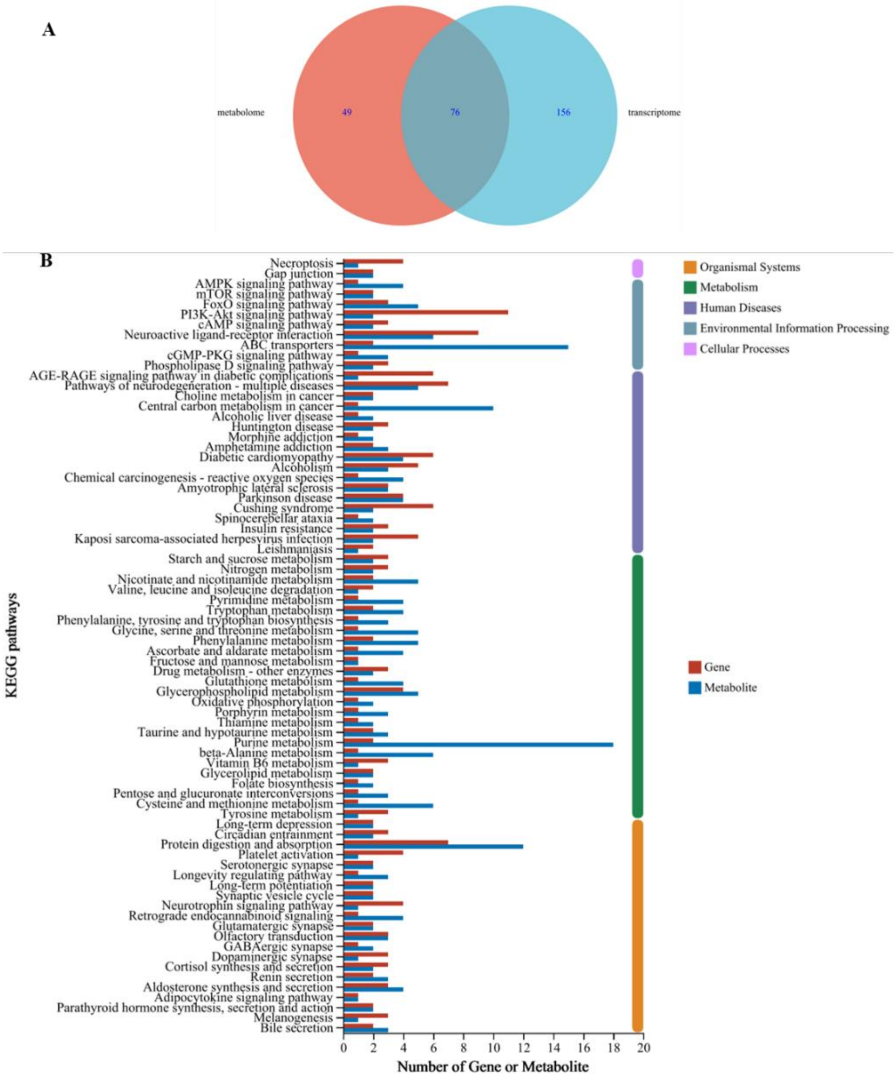

Figure S2　Annotated Venn and bar plots of differential genes and differential metabolite KEGG enrichment pathways in the 39 ℃ HS-CON group

Note: Figure A shows the Venn diagrams of pathways annotated in the gene set and pathways annotated in the metabolic set; The horizontal axis in B represents the number of genes or metabolites in the pathway, where the blue band represents the number of genes annotated into the pathway, the red band represents the number of metabolites annotated into the pathway, and the vertical axis represents the KEGG pathway name. The annotations for the same type of diagram in the following text are the same as this annotation.

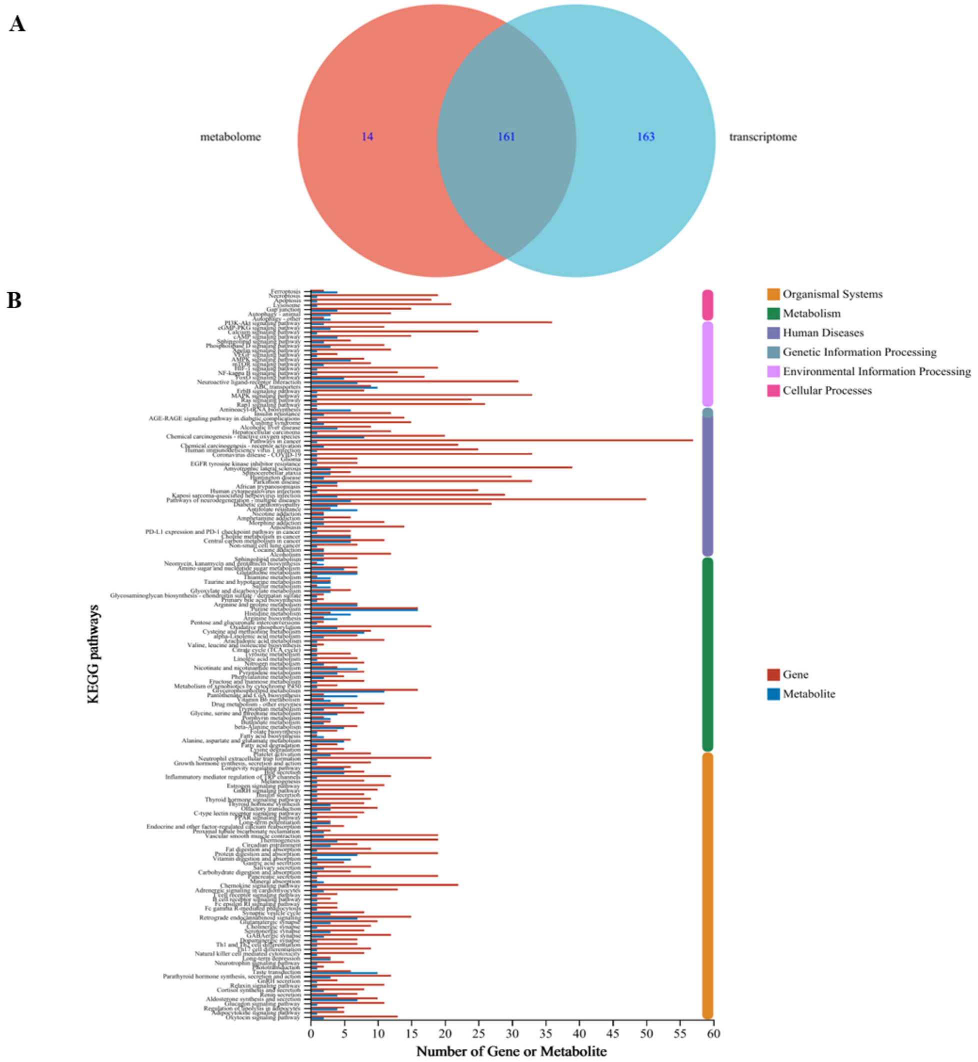

Figure S3 Annotated Venn and bar plots of differential genes and differential metabolite KEGG enrichment pathways in the 41 °C HS-CON group

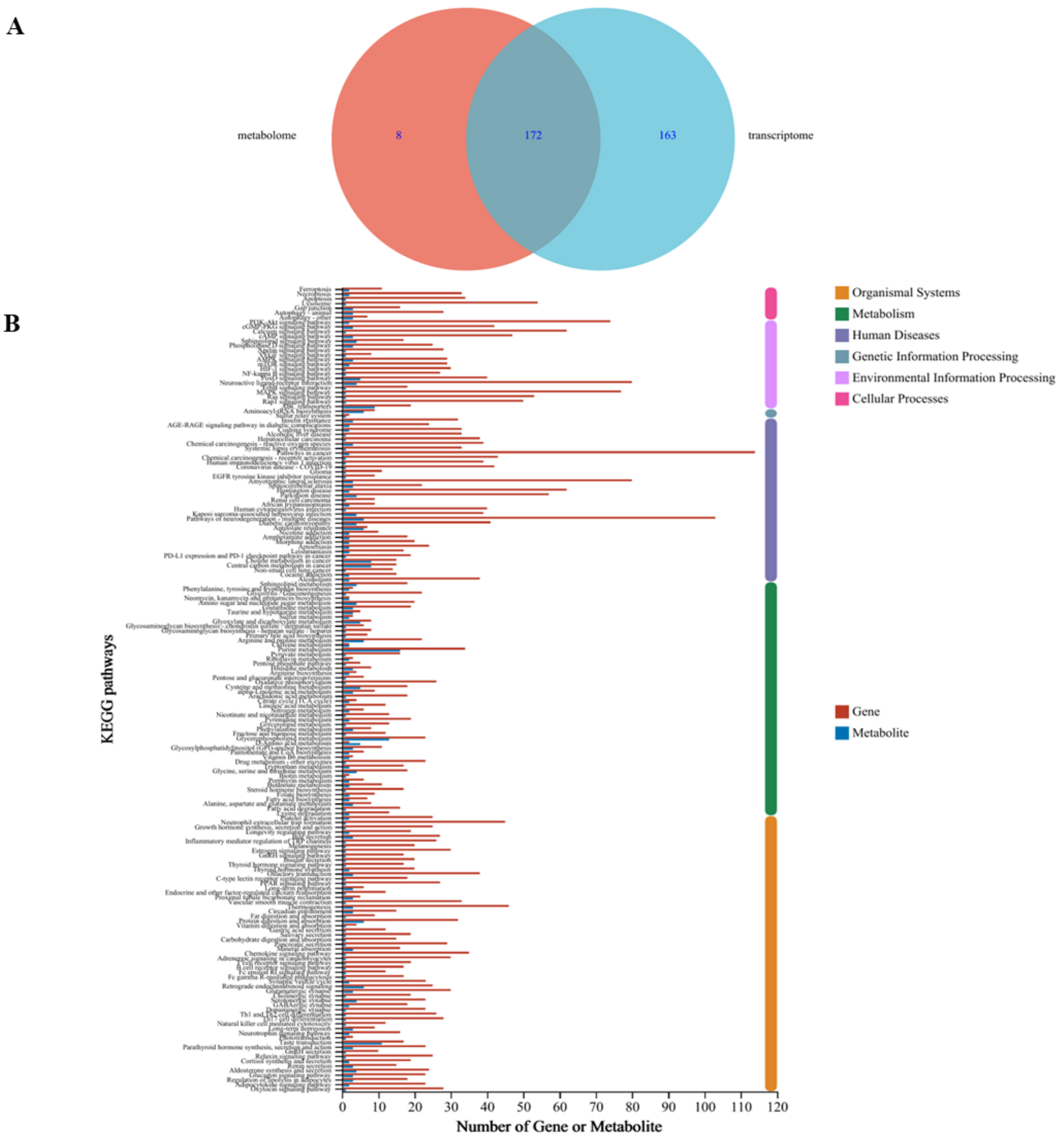

Figure S4 Annotated Venn and bar plots of differential genes and differential metabolite KEGG enrichment pathways in the 43 °C HS-CON group
